# Disentangling the impact of obesity, diet, host factors, and microbiota on small intestinal antimicrobial peptide expression

**DOI:** 10.1080/19490976.2025.2536095

**Published:** 2025-08-04

**Authors:** Fabiola Puértolas-Balint, Vishnu Prasoodanan P K, Sandra M. Holmberg, Bjoern O. Schroeder

**Affiliations:** a Department of Molecular Biology, Umeå University, Umeå, Sweden; b The Laboratory for Molecular Infection Medicine Sweden (MIMS), Umeå University, Umeå, Sweden; c Umeå Center for Microbial Research (UCMR), Umeå University, Umeå, Sweden

**Keywords:** Antimicrobial peptides, high-fat diet, metabolic disease, small intestinal microbiota, Western-style diet

## Abstract

The small intestinal mucosa has the delicate task of allowing absorption of nutrients and limiting microbial colonization at the mucosal surface through production of antimicrobial peptides and proteins (AMPs). However, while environmental factors, including different diets, have been shown to alter AMP expression, the results from the literature are conflicting on their specific impact. Moreover, the interdependence between diet, AMPs, and metabolic health is largely unexplored. The aim of this study was thus to investigate the effect of obesogenic diets, obesity itself, and other variables, including mouse vendor, microbiota composition, and sex, on intestinal AMP expression. By using different dietary interventions in mice, we here show that prolonged intake of an obesogenic Western-style diet had a stronger impact on AMP expression than diet-independent obesity. Additionally, when comparing AMP expression under different diets in mice of both sexes from different vendors, the combined contribution of these factors had the strongest impact on absolute AMP transcript numbers, but also on the variability in small intestinal microbiota composition at the mucosa and content. Finally, we identified a novel host–microbe interaction, in which the gut commensal *Faecalibaculum* bloomed upon WSD-feeding and specifically induced the expression of the AMP Reg3g. Our findings thus reveal that the experimental setup, defined by mouse vendor, sex, and diet type, has a major influence on small-intestinal AMP expression. These findings could partly explain the discrepancy in the literature regarding the effect of diets on AMP expression, preventing any accurate generalization about the impact of diet on the antimicrobial response.

## Introduction

The human intestine has the pivotal role in nutrient digestion and absorption. While lipids, amino acids, and simple carbohydrates are digested and absorbed in the small intestine, water and electrolytes are absorbed in the large intestine. Nutrient digestion is facilitated by the intestinal bacterial microbiome: in the small intestine, gut bacteria conjugate bile salts, stimulate bile-inducing hormones,^1^ and can influence lipid and glucose sensing,^2^ while in the large intestine they degrade complex nondigestible dietary fibers.^1^

Despite their beneficial role, the bacterial members of the microbiome need to be kept at a safe distance from the intestinal epithelium to prevent an undesired infection and inflammatory response. Hence, the host has evolved specific regio-specific functional mechanisms to protect the mucosa. In the small intestine, a gel-like mucus layer serves as a physical protection and harbors a defined commensal microbial community to prevent infection,^3^ while the secretion of various families of antimicrobial peptides and proteins (AMPs) serves as a biochemical layer of defense.^4–7^

Small intestinal antimicrobial peptides are primarily produced by Paneth cells. They are small cationic peptides, typically between 20 and 40 amino acids for α-defensins (also known as cryptdins in mice) or β-defensins, which are bactericidal by forming pores in bacterial cell membranes.^8,9^ Other antimicrobial proteins, such as human defensin 6 (HD6), Ly6/PLAUR domain containing 8 (Lypd8) and Zymogen Granule Protein 16 (ZG16), act by entrapping bacteria and thereby immobilizing them.^10–12^ While α-defensins are constitutively expressed, studies in germ-free (GF) mice have suggested that the microbiota is required for the full induction of specific AMPs, including α-defensins, cryptdin-related sequence peptides (CRS-peptides), secreted phospholipase A2 (sPLA2a), Resistin-like molecule beta (RelmB), as well as the antimicrobial proteins Lysozyme (P-Lys) and Regenerating islet-derived protein 3 gamma (Reg3g).^8,13,14^ As such, supplementation of probiotic bacteria in humans^15^ and mice^16,17^ could lead to increased expression of human beta defensin-2 (HBD2) and Reg3g, respectively. Moreover, bacterial molecules such as lipopolysaccharide (LPS) and membrane components have been shown to induce AMP secretion in isolated Paneth cell-containing crypts from mice.^18^ Consequently, the presence of bacteria or bacterial products leads to increased AMP expression, and in turn, AMPs neutralize bacteria that reach too close proximity to the intestinal mucosa, thereby maintaining a fine-tuned homeostasis in the intestinal mucosa.

Disturbances in the mucosal homeostasis through alterations in defensin levels have been linked to several life-style associated diseases, including the inflammatory bowel diseases (IBD) Crohn´s disease and ulcerative colitis,^19,20^ as well as metabolic diseases such as obesity.^21^ While these diseases are complex and multifactorial, the consumption of Western-style diets (WSD), high in fat and simple sugars but lacking dietary fiber, has been associated with disease progression both in IBD and obesity.^22^ Interestingly, the effect of diet on AMP expression in mice is conflicting in the literature: high-fat diets have been shown to reduce^17,23–26^ or increase their expression,^27,28^ and the reason for the contrasting results across studies is not fully understood. While biological reasons may be behind these observations, different experimental set-ups and analyses could also play an important role.^29^ Indeed, the regulation of AMP expression is complex and has been shown to be modulated by diet and the microbiota.^14,29–31^ In addition, a study solely using female mice showed that supplementation with inulin under a WSD, recovered the otherwise decreased AMP expression.^26^ Thus, factors that could influence the dietary modulation of AMP expression include diet type, different microbiota composition, and sex.

The link between AMPs and metabolism is receiving increased attention. Patients with obesity produce lower amounts of HD5 and Lysozyme in the small intestine,^21^ and induction of Reg3g expression following vertical sleeve gastrectomy or probiotic supplementation has been linked to improvements in glucose and insulin homeostasis in mice.^17^ The effect of Reg3g in glucose modulation was confirmed by supplementing this AMP to mice feeding on a 60% high fat diet (HFD), which resulted in improved oral glucose tolerance.^17^ Likewise, administration of HD5 to HFD-fed mice improved the diet-dependent worsening in glucose and insulin tolerance.^32^ Therefore, this evidence suggests that AMPs help control metabolic dysfunction in mice.

In previous work, we observed that mice fed a WSD (40% fat, 40% carbohydrate diet) and that lacked active α-defensins had increased body fat, insulin levels, and HOMA-IR when compared to wild-type mice that were fed the same high caloric diet.^7^ This defensin-dependent difference was only observed in mice fed a WSD but not in mice fed a chow control diet, highlighting a complex interdependence between metabolic parameters and small intestinal α-defensins, which warrants further investigation. Thus, we here aim to disentangle the effect of obesogenic diet, obesity itself and other variables on intestinal AMP expression.

## Materials and methods

### Mice and ethics approval

**Figure 1.**
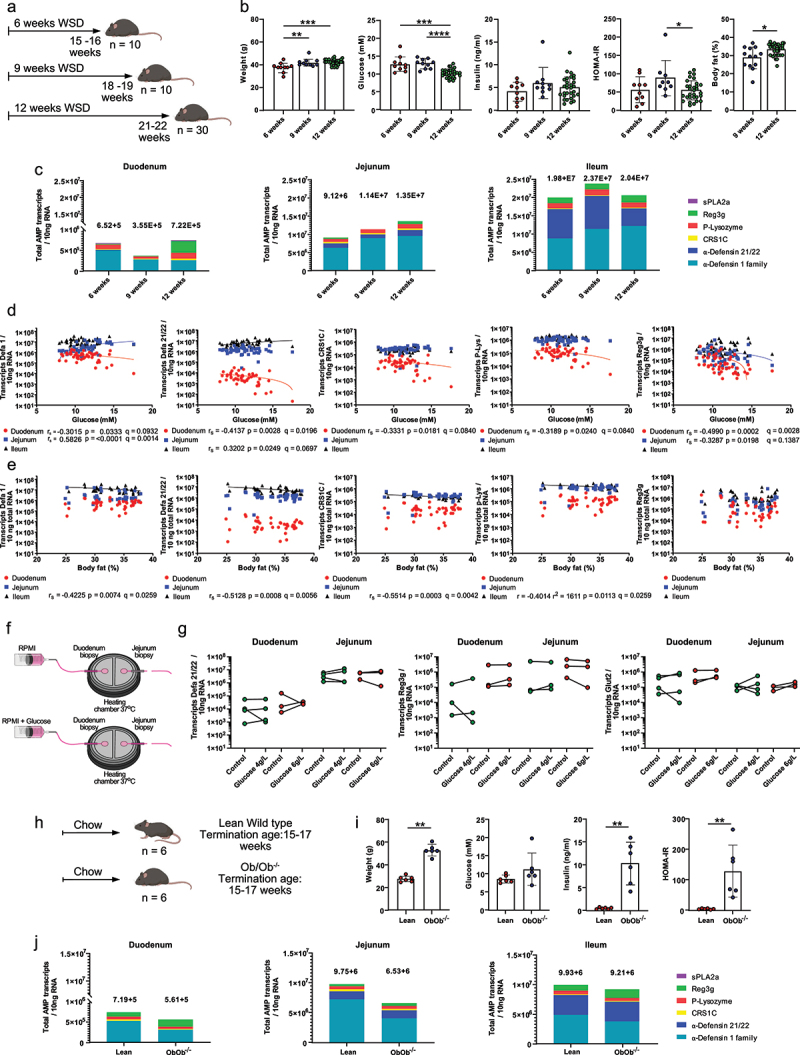
AMP expression is moderately affected by prolonged diet-induced obesity but not in genetic obesity.

**Figure 2.**
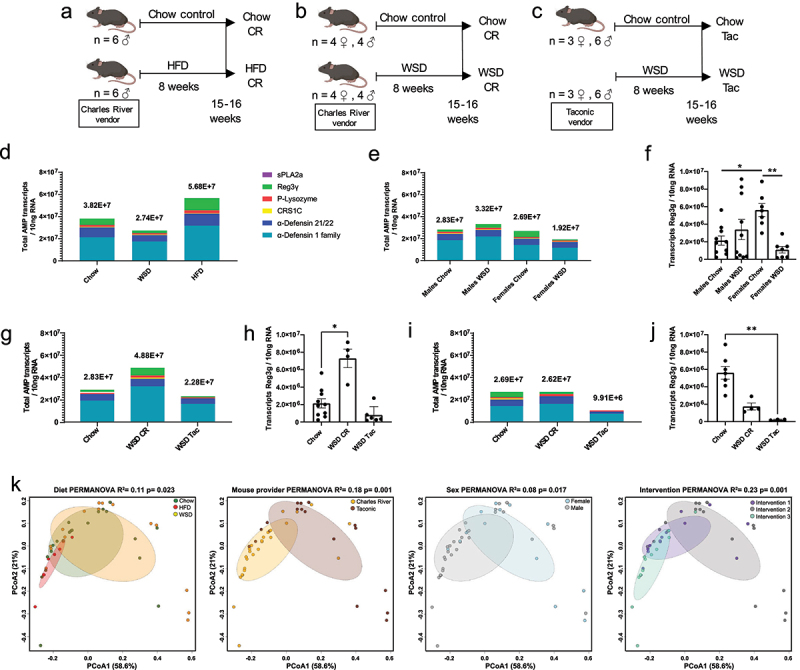
Different factors can impact AMP expression in the murine small intestine.

C57BL/6J mice included in this study were housed in up to 4–5 mice per cage under specific pathogen-free conditions (SPF), with unlimited access to water and food. Mice from the experiment that evaluated AMP expression in mice groups with different durations of Western-style diet (WSD) feeding (Figure 1(a)) were bred in-house while leptin deficient *Ob/Ob*^−/−^ mice were originally obtained as heterozygous pairs from Charles River, Italy, and bred in-house for several generations to obtain littermate-controlled *Ob/Ob*^−/−^ and *Ob/Ob*^+/+^ mice (Figure 1(h)). Male and female *Ob/Ob*^−/−^ and lean wild-type littermate mice were housed in separate cages. The remaining dietary interventions (Figure 2(a-c)) were carried out with wild-type C57BL/6J mice purchased from Charles River or Taconic farms and allowed for acclimatization to the facility for 3 weeks. Apart from the WSD-duration experiment (Figure 1(a)), all mice with a diet intervention were age matched (15–17 weeks) when sacrificed by anesthesia with isofluorane and cervical dislocation.

(a) Schematic experimental setup of the effect of WSD duration on AMP expression: Groups of mice were fed a Western-style diet (WSD) for 6, 9 and 12 weeks. (b) Metabolic parameters: bodyweight, fasting blood glucose and insulin concentration, Homeostatic Model Assessment for Insulin Resistance (HOMA-IR), and body fat of the mice from the WSD duration experiment. (c) Total amounts of all measured AMP transcripts in the duodenum, jejunum, or ileum from the WSD duration experiment. (D,E) Expression of individual AMPs at the duodenum (red), jejunum (blue) and ileum (black) of the WSD duration experiment, was correlated with blood glucose levels (d) and with (e) body fat. Pearson (normally distributed data) or Spearman (non-normally distributed data) correlation analysis was used to test correlations between AMP expression and metabolic parameters. (f) Scheme of *ex-vivo* incubation of mouse biopsies obtained from duodenum or jejunum with and without addition of glucose. (g) Absolute expression of *Defa21/22*, *Reg3g* and *Glut2* of small intestine biopsies incubated *ex-vivo* with RPMI or RPMI supplemented with glucose. (h) Schematic experimental set-up of the genetic obesity experiment using littermate-controlled lean wild-type (WT) and *Ob/Ob*^−/−^ mice fed a chow diet. (i) Metabolic parameters: bodyweight, fasting blood glucose and insulin concentration and HOMA-IR of the mice from the genetic obesity experiment. (j) Total amounts of all measured AMP transcripts in the duodenum, jejunum, or ileum from the genetic obesity experiment. Statistically significant correlations are indicated by colored lines and correlation coefficients with *p*- and q-values are shown. Mann-Whitney U test was used to determine statistical significance in panels B,G (compared to control section coming from the same mouse) and I. * = p < 0.05, ** = p < 0.01, *** = p < 0.001, **** = < 0.0001 were considered statistically significant.

(a, b, c) Schematic experimental setup of the dietary intervention experiments: (a) Intervention 1, male Charles River mice fed a chow or a high-fat diet (HFD) for 8 weeks; (b) Intervention 2, male and female Charles River mice fed a chow or a Western-style diet (WSD) for 8 weeks; (c) Intervention 3, male and female Taconic mice fed a chow or a WSD for 8 weeks. (d) Total amounts of all measured AMP transcripts in the ileum of all mice fed a chow, WSD, or HFD. (e) Total amounts of all measured AMP transcripts in the ileum of male and female mice fed a chow or WSD from interventions 2 and 3. (f) Reg3g transcript copy number in the ileum of chow and WSD fed mice from interventions 2 and 3. (g) Total amounts of all measured AMP transcripts in the ileum of male Charles River or Taconic mice fed a chow or a WSD. (h) Reg3g transcript copy number in the ileum of male Charles River or Taconic mice fed a chow or a WSD. (i) Total amounts of all measured AMP transcripts in the ileum of female Charles River or Taconic mice fed a chow or a WSD. (j) Reg3g transcript copy number in the ileum of female Charles River or Taconic mice fed a chow or a WSD. (k) Principal component analysis (PCoA) evaluating the effect of diet, mouse vendor, sex, or intervention on AMP expression in mice from the dietary interventions. Statistical tests were calculated with a 2-way ANOVA with Tukey’s Multiple comparisons for Reg3g transcript quantification between sexes (f), One-Way ANOVA with Dunn’s multiple comparison for Reg3g transcript quantification in different vendors (h, j) and with a PERMANOVA test for PCoA analysis (k). * = p < 0.05, ** = p < 0.01, *** = p < 0.001, ****= < 0.0001 were considered statistically significant.

The WSD-duration of AMP expression (Figure 1(a)) and *Ob/Ob*^−/−^ (Figure 1(h)) mouse experiments were carried out and approved by the local animal authority at the University of Gothenburg, Sweden. Subsequent experiments with Charles River mice fed a chow or HFD (dietary intervention 1, Figure 2(a)), Charles River mice fed a chow or WSD (dietary intervention 2, Figure 2(b)), Taconic mice fed a chow or WSD (dietary intervention 3, Figure 2(c)), and mice from our own breeders (originally Charles River) used for *ex-vivo* incubation (Figure 1(f)) or infected with bacteria (Figure 6(c)) were carried out and approved by the local animal authority at Umeå University, Sweden.

### Mouse diets

Mice from the groups that received WSD for 6, 9 or 12 weeks (Figure 1(a)) and Male and female Charles River and Taconic mice (Figure 2(b,c)) from the 8 week intervention were fed a WSD (TD.96132 or TD.09683 Envigo: 40.6% kcal from fat, 40.7% from carbohydrates (sucrose 18.2% (w/v), corn starch 16.0% (w/v), maltodextrin 12.0% (w/v), cellulose 4.0% (w/v)).

Lean and *Ob/Ob*^−/−^ (Figure 1(h)) were fed with a standard chow diet (5021 LabDiet: 23.7% kcal from fat, 53.2% from carbohydrates (sucrose 0.71% (w/v), starch 31.0% (w/v) glucose 0.21% (w/v), neutral detergent fiber 15.2% (w/v)).

Male and female Charles River and Taconic mice (Figures 2(a–c) and 6(a)) were fed a standard chow diet SDS 801,730 CRM expanded: 9.12% kcal from fat, 68.68% from carbohydrates (pectin 1.4% (w/v), hemicellulose 8.82% (w/v), cellulose 3.85% (w/v), lignin 1.4% (w/v), starch 42.63% (w/v), sugar 3.94% (w/v), total dietary fiber 14.99% (w/v)) for 8 weeks.

Charles River male mice (Figure 2(a)) were fed a high-fat diet (HFD) ssniff 60 kcal% fat – Tallow low sucrose: 61% kcal from fat, 20% from carbohydrates (sucrose 2.9% (w/v), starch 12.7% (w/v), dextrin 7.9% (w/v), sugar 3.9% (w/v), crude fiber 6% (w/v)) for 8 weeks.

### Metabolic characterization

To measure blood glucose levels, mice were fasted for 4 h, and glucose concentration was measured from tail vein blood with commercial blood glucose strips (Contour Next, Bayer). Insulin concentration in blood was determined in serum with the Ultra Sensitive Mouse Insulin ELISA kit (Crystal Chem). Body fat was determined by whole-body magnetic resonance imaging (EchoMRI, Echo Medical Systems). The homeostatic model assessment insulin resistance index (HOMA-IR) was determined with the formula HOMA-IR = (insulin (mU/L) * glucose (mM))/22.5.

### RNA extraction and cDNA synthesis

The small intestine was dissected, divided into eight equal parts and numbered from proximal to distal segments. The first segment is considered as the duodenum, the fifth segment is considered to represent the jejunum, and the eighth represented the ileum. Mouse tissue from duodenum, jejunum, and ileum were homogenized with stainless steel beads (5 mm) (Qiagen) in a TissueLyser II (Qiagen), and RNA was extracted using a RNeasy mini kit (Qiagen). The RNA quantity and quality were assessed by NanoDrop (Thermo Fisher Scientific). A total of 500 ng of RNA was reverse transcribed into cDNA with a High-Capacity cDNA Reverse Transcription kit (Applied Biosystems, Thermo Fisher Scientific) and diluted 1:7 in nuclease-free water.

### Transcript copy number determination by qPCR

Plasmids containing AMP-specific cDNA sequences generated previously^14^ and a *Glut2* plasmid generated in this study, were used to generate a standard curve in a 10-fold dilution series. Duodenal, jejunal, and ileal cDNA samples were analyzed as duplicates in a 10-µL reaction mix consisting of 1× iQ SYBR Green Supermix (Bio-Rad), 0.2 mM each primer, and 2 µL of template cDNA on a CFX Connect Real-Time System (Bio-Rad). Amplification of samples and plasmid standards was performed as follows: denaturation at 95°C for 3 min, followed by 35 cycles of denaturation at 95°C for 20 s, gene-specific annealing temperature (Table 1) for 40 s, and extension at 72°C for 60 s. A master standard curve was used to determine copy number of genes across experiments, and expression is reported as copy number/10 ng RNA.

**Table 1.** Primer sequences used in this study and annealing temperature used for qPCR.

Name	Product	Primer sequence		Annealing temp.
Alpha defensin 1 family	Defa1	Fwd (5’-3’)	TCAAGAGGCTGCAAAGGAAGAGAAC	63°C
		Rvs (5’-3’)	TGGTCTCCATGTTCAGCGACAGC	
Alpha defensin 21/22	Defa21/22	Fwd (5’-3’)	CCAGGGGAAGATGACCAGGCTG	63°C
		Rvs (5’-3’)	TGCAGCGACGATTTCTACAAAGGC	
Cryptdin related sequence (CRS) peptides Group 1C	CRS1C	Fwd (5’-3’)	CACCACCCAAGCTCCAAATACACAG	68°C
		Rvs (5’-3’)	ATCGTGAGGACCAAAAGCAAATGG	
Reg III gamma	Reg3g	Fwd (5’-3’)	CCTCAGGACATCTTGTGTCTGTGCTC	68°C
		Rvs (5’-3’)	TCCACCTCTGTTGGGTTCATAGCC	
Paneth cell specific lysozyme	P-Lys	Fwd (5’-3’)	GCCAAGGTCTACAATCGTTGTGAGTTG	66°C
		Rvs (5’-3’)	CAGTCAGCCAGCTTGACACCACG	
Secretory phospholipase A2	sPLA2a	Fwd (5’-3’)	AGGATTCCCCCAAGGATGCCAC	68°C
		Rvs (5’-3’)	CAGCCGTTTCTGACAGGAGTTCTGG	
Glucose transporter 2	*Glut2*	Fwd (5’-3’)	TTCCAGTTCGGCTATGACATCG	60°C
		Rvs (5’-3’)	CTGGTGTGACTGTAAGTGGGG	
*Faecalibaculum rodentium*	*F. rodentium*^47^	Fwd (5’-3’)	CCGGGAATACGCTCTGGAAA	60°C
		Rvs (5’-3’)	GCCAACCAACTAATGCACCG	

To determine the copy number of *Faecalibaculum rodentium* in mucosa samples, mucosa-associated bacteria (MAB) DNA was analyzed in a 10 μl reaction mix consisting of 1× qPCRBIO SYGreen mix (Techtum), 0.2 μM of each forward and reverse *F. rodentium* primer (Table 1) and 2 μl of MAB DNA on a CFX Connect Real-Time System (Bio-Rad). Duplicates of samples were amplified by using the following protocol: denaturation at 95°C for 3 min, followed by 35 cycles of denaturation at 95°C for 15 s, annealing at 60°C for 30 s, and extension at 72°C for 30 s. A standard curve with *F. rodentium*-specific plasmids was prepared, and the *F. rodentium* copy number in each sample was calculated using Bio-Rad CFX Maestro software. The results are reported as *F. rodentium* copy number/ng of total MAB DNA extracted from each biopsy.

### Ex vivo small intestinal biopsy incubation

The duodenum or jejunum of male and female C57BL/6J mice bred at the local animal facility, were gently flushed with ice-cold Kreb’s buffer (116 mM NaCl, 1.3 mM CaCl_2_, 3.6 mM KCl, 1.4 mM KH_2_PO_4_, 23 mM NaHCO_3_, and 1.2 mM MgSO_4_ (pH 7.4)) to remove luminal content. After removal of the muscle layer, duodenum, or jejunum sections coming from the same mouse were cut into two pieces and mounted into two separate perfusion chamber systems, one supplemented with a continuous basolateral supply of RPMI and the other with RPMI supplemented with glucose for a final 4 g/L or 6 g/L. The tissues were covered in phosphate buffer saline (PBS) in the apical side to avoid drying. The chambers were heated to 37°C in heating blocks, and the duodenum and jejunum tissues in two separate chambers were incubated simultaneously for 1 hour (Figure 1(f)). The tissue biopsies were frozen and kept at −80°C until RNA extraction. The resulting gene expression following supplementation of RPMI with glucose of the duodenum and jejunum biopsies was compared to the RPMI control biopsies from the same site.

### DNA extraction and sequencing of small intestinal bacteria from the ileal lumen and mucosa

For 16S gut bacterial analysis, the intestinal content was collected (luminal sample), and the tissue was cut longitudinally and washed twice in Mg-free PBS to remove any leftover content attached to it (mucosa sample). The content or mucosa samples were frozen in liquid nitrogen and stored at −80°C. Genomic DNA from mucosal tissue and intestinal lumen content was extracted by repeated bead-beating with a lysis buffer (4% [wt/vol] SDS, 50 mM Tris HCl pH 8, 500 mM NaCl, 50 mM EDTA) in a Fast-Prep System with Lysing Matrix E (MPBio) as described previously,^33^ and purified with a QIAmp DNA mini kit (Qiagen).

The extracted and purified DNA was submitted for library preparation for 16S rRNA amplicon sequencing on an Illumina MiSeq machine with a V2 kit (2 × 250 bp paired-end reads). Briefly, the V4 region of the 16S rRNA gene was amplified using 515F and 806 R primers designed for dual indexing.^34^ The amplification was controlled for purity with a non-template control for each sample. Lumen content and MAB tissue samples were amplified in triplicates or quadruplicates, in a reaction volume of 25 µL containing 100 ng of genomic DNA, 1x Five Prime Hot Master Mix (Quantabio), 0.2 mM final of each primer, 0.4 mg/mL bovine serum albumin (BSA) and 5% DMSO. PCR amplification was performed as follows: initial denaturation at 94°C for 3 min, 27 cycles (MAB) or 26 cycles (content) of denaturation at 94°C for 45 s, annealing at 52°C for 60 s and elongation at 72°C for 90 s, and a final elongation step at 72°C for 10 min. The PCR products were pooled and purified using the NucleoSpin Gel and PCR Clean-Up kit (Macherey-Nagel), quantified (Quant-iT PicoGreen dsDNA kit; Thermo Fisher Scientific), and pooled to equimolar amounts. The pooled 16S amplicons were further purified using Mag-Bind magnetic purification beads (Omega Biotek) before denaturation of the libraries for loading into the Illumina V2 cartridge following sequencing on an Illumina MiSeq system.

### Sequencing analysis of small intestinal bacteria from lumen and mucosa

We obtained paired-end reads from 91 samples, totaling 8,937,168 (median 72,159 reads per sample). Initial quality check was conducted using the FASTQC tool^35^ available online at: http://www.bioinformatics.babraham.ac.uk/projects/fastqc. Low-quality reads were filtered out utilizing the FastX toolkit (http://hannonlab.cshl.edu/fastx_toolkit/index.html.) with criteria set at a minimum quality score of 25 and a minimum of 70% of bases having a quality score of 25. Subsequently, another quality check was performed post-filtration. To ensure paired-end reads remained intact after filtration, we used the repair.sh tool from the Bbmap package to remove any single-end reads (paired-end reads that lost their pair during quality filtration). Two samples with fewer than 500 sequences were excluded from further analysis. The number of reads remaining after quality filtration ranged from 2,460 to 317,355 in each sample, excluding the aforementioned samples.

Further analysis of 16S amplicon sequences was performed using the QIIME2^36^ (version 2021.4) pipeline and R (version 4.1.3) in R Studio (RStudio Team, version 2022.07.2). Sequences were then clustered into ASVs using the SILVA classifier (version 138).^37^ The data were further filtered to remove mitochondria and chloroplasts, and specific *Lactococcus lactis* ASVs were removed since it is a known contaminant in the WSD pellets.^38^ DADA2 was employed to generate the feature table, which comprises ASV-IDs along with the corresponding count of detected ASVs in each sample.^39^ Using the ASV inclusion criterion that an ASV should be present in a minimum of two samples with a total ASV count of 10. Four hundred and sixty-nine ASVs were excluded based on their low abundance. Following the ASV inclusion criterion, we retained the abundance data of 1,165 ASVs for subsequent analysis.

Diversity measures were evaluated using the relative abundance of 1,165 ASVs in each sample. β-diversity was assessed using genus counts subjected to a center log ratio (CLR) transformation. The ASV count per taxonomic clade per sample was normalized by dividing the ASV count by the total number of reads in the corresponding sample to generate the relative abundance. Transformation of genus and phylum abundance data into CLR-transformed values was carried out by using the taxa_transform function from the phyloseq package.^40^ The α-diversity metrics, including observed species and Shannon index, and β-diversity, represented by Aitchison distance (Euclidean distance calculated on species counts subjected to a center log ratio transformation), were calculated using phyloseq, vegan (https://cran.r-project.org/web/packages/vegan/vegan.pdf), and ape (https://cran.r-project.org/web/packages/ape/ape.pdf). Principal coordinate analysis (PCoA) was carried out using Aitchison distance between samples based on their CLR-transformed genus abundances. The number of reads assigned to different taxonomic classes (mainly phylum and genus) was calculated, and the taxonomic composition was evaluated for each sample using both relative abundance and CLR-transformed ASV counts. Statistical differences between groups were calculated by using Kruskal – Wallis or PERMANOVA and 999 permutations (β -diversity).

### Correlation analysis

Absolute AMP copy numbers measured in duodenum, jejunum, and ileum from the mice groups that were fed WSD for 6, 9 or 12 weeks were correlated with the metabolic parameters blood glucose, blood insulin, bodyweight, and body fat. The normal distribution of the data was tested with a D’Agostino & Pearson test, and normally distributed data were tested with a Pearson test and non-normally distributed data were tested with a Spearman ranks test, and respective r or r_s_ correlation rank was reported. The plots were generated using GraphPad Prism (version 10).

Log-transformed AMP transcript copy numbers and the relative abundance of microbial genera were used to assess correlations between microbiome composition and AMP expression. Associations were identified using HAllA,^41^ which is designed for robust correlation analysis in heterogeneous datasets. Results were visualized as a HAllAgram, where block associations are ranked by significance, with each block representing a cluster of co-occurring microbial genera associated to a group of AMPs. White dots indicate marginally significant pairwise associations. Multiple testing correction was applied using the Benjamini–Hochberg–Yekutieli (BHY) procedure.

### Faecalibaculum rodentium infection

*Faecalibaculum rodentium* DSM 103405 was cultured in Gifu anaerobic medium (GAM) (HyServe) in an anaerobic chamber (Don Whitley Scientific, UK) at 37°C overnight. Aliquot stocks of the bacterium in fresh GAM media with a final 15% glycerol were stored at −80°C.

Male and female C57BL/6 mice bred at the local animal facility, aged 8–11 weeks old were kept on a chow diet, and were orally gavaged with either *F. rodentium* or reduced PBS (0.1% L-cysteine). Prior to infection, 1 ml bacteria stocks were centrifuged at 4000 × g for 5 min and the bacterial pellet was resuspended in anaerobic reduced (0.1% L-cysteine) PBS. Each mouse received 200 µl per gavage (containing ~3 × 10^8^ CFUs) 3 times per week for 3 weeks.

### Statistical evaluation

For comparisons of non-parametric data between two groups, the Mann-Whitney U test was used. For comparisons among more than two groups, the Kruskal-Wallis one-way ANOVA or the 2-way ANOVA with Tukey’s post hoc test was employed. To compare multiple treatments with a control treatment, a one-way ANOVA with Dunn’s multiple correction was utilized. A PERMANOVA analysis was used for PCoA analysis of AMP expression. β-diversity analysis between groups was estimated with Euclidian distances and statistical differences between groups were calculated by using Kruskal – Wallis or PERMANOVA and 999 permutations. HAllA was used to identify significant correlations between genus-level taxonomic composition and AMP expression profiles using Spearman correlation values. All the tests were performed in GraphPad Prism (version 10) unless mentioned otherwise in the methods.

The concordance between microbiome relative abundance and log-transformed AMP expression data was assessed using Procrustes analysis (vegan package https://cran.r-project.org/web/packages/vegan/vegan.pdf), based on Euclidean inter-sample distances. Ordinations were performed using PCoA (microbiome and AMP) , and the congruence between datasets was quantified using the sum of squared distances between matched sample points after optimal superimposition (m12^2^), with statistical significance assessed via permutation (*n* = 999) testing. Visualization of the Procrustes alignment was done using ggplot2 (https://ggplot2.tidyverse.org/.), gridExtra (https://cran.r-project.org/web/packages/gridExtra/index.html.), and ggrepel (https://cran.r-project.org/web/packages/ggrepel/index.html). Mantel tests (vegan) were used to evaluate correlations between distance matrices.

### Data availability

Amplicon sequences have been deposited in the European Nucleotide Archive (https://www.ebi.ac.uk/ena/) with accession number PRJEB88508.

## Results

### Diet-induced obesity, but not genetic obesity, modulates Paneth cell AMP expression in a regio-specific manner

In mouse models of diet-driven obesity, the change in diet will result in changes in microbiota composition, which is expected to affect AMP expression.^28,42^ To thus rule out the effect of any potential diet-driven microbiome changes in AMP expression, and to focus on the effect of metabolic dysfunction on AMP expression, we separately fed wild-type mice groups a high-fat/low-fiber containing Western-style diet (WSD) for 6, 9 and 12 weeks (Figure 1(a)), a time window in which diet-driven changes in small-intestinal microbiota composition have already occurred.^3,42^ As expected, the metabolic evaluation of these mice showed a gradual increase in body weight, where the weight difference was significantly increased after 9 or 12 weeks compared to 6 weeks (*p* = 0.0073, *p* = 0.0039) (Figure 1(b)).

Strikingly, blood glucose levels decreased after 12 weeks of WSD-feeding when compared to 6 or 9 weeks (*p* = 0.0039, *p* < 0.0001) (Figure 1(b)), indicating a potential adaptation of host metabolism to the WSD and metabolic alterations. While no significant difference in insulin levels was observed at the different time-points, the homeostatic model assessment of insulin resistance (HOMA-IR) identified a significant decrease after 12 weeks when compared to 9 weeks (*p* = 0.0205), following the blood glucose levels. Nevertheless, mice feeding on a WSD for 12 weeks had increased body fat (*p* = 0.0150) when compared to mice fed a WSD for 9 weeks (Figure 1(b)).

To determine the effect of the diet-induced metabolic dysfunction on AMP expression, we analyzed the expression of Paneth cell-specific AMPs in the duodenum, jejunum, and ileum for the different time points (Figure 1(c)) using absolute transcript quantification instead of relative expression. Mice fed a WSD for 9 weeks had slightly lower total AMP copy numbers in the duodenum than mice fed for 6 or 12 weeks (Figure 1(c)), mainly driven by significantly lower levels of Defa 21/22 transcript copy number (*p* = 0.0355 and *p* < 0.0001, respectively; Supplementary Figure S1a). In addition, after 12 weeks of WSD consumption, the duodenal levels of Defa 1-family (*p* = 0.0624), P-Lys (*p* = 0.0003), CRS1C (*p* < 0.0001), and Reg3g (*p* < 0.0001) were significantly higher than after 9 weeks of WSD-feeding (Supplementary Figure S1a).

In the jejunum, we observed a moderate increase in total AMP expression from 6 to 12 weeks of WSD-feeding (Figure 1(c)), whereby mice fed a WSD for 12 weeks had significantly higher levels of Defa 1-family (*p* = 0.0283), P-Lys (*p* = 0.0283), Reg3g (*p* = 0.0394), and sPLA2a (*p* = 0.0260) when compared to mice that were fed the WSD for 6 weeks (Supplementary Figure S1b). Likewise, a strong increase in Reg3g expression was even observed between 9 weeks and 12 weeks of WSD-feeding (*p* = 0.0002) (Supplementary Figure S1b).

In contrast to the duodenum, the total AMP copy numbers of the ileum were moderately higher in mice fed a WSD for 9 weeks when compared to mice fed for 6 or 12 weeks (Figure 1(c)), driven by higher levels of Defa 21/22 (*p* = 0.0058), CRS1C (*p* = 0.0037), sPLA2a (*p* = 0.0397) when compared to 12 week fed mice, and higher levels of sPLA2a (*p* = 0.0185) when compared to 6 week fed mice (Supplementary Figure S1c).

Consequently, these findings suggest that AMP expression in the small intestine responds to prolonged WSD treatment in a regio-specific manner, with distinct AMPs having opposite directions of response between the time points and regions. For example, the expression of Defa21/22 and CRS1C increased after 12 weeks when compared to 9 weeks in the duodenum, but it decreased in the ileum between these time points. Reg3g expression also increased after 12 weeks when compared to 9 weeks in the duodenum and jejunum, but the average expression levels remained unchanged between 6, 9 and 12 weeks in the ileum.

Based on the influence of diet, we next aimed to determine the specific associations between the diet-affected metabolic parameters with AMP expression in different regions of the small intestine. We thus correlated the expression of individual AMPs with body weight, blood glucose, insulin, HOMA-IR and body fat from the mice groups fed a WSD for different durations. While most correlation analyses yielded no significant associations, there was a consistent negative correlation between the expression of individual AMPs and blood glucose in the duodenum (Defa 1-family *r* = −0.3015 *p* = 0.0333, Defa21/22 r = −0.4137 *p* = 0.0028, CRS1C *r* = −0.3331 *p* = 0.0181, P-Lys *r* = −0.3189 *p* = 0.0240, Reg3g *r* = −0.4990, *p* = 0.0002), as well as for Reg3g in the jejunum (*r* = −0.3287, *p* = 0.0198) (Figure 1(d)). Additional significant positive correlations with blood glucose included Defa 1-family in the jejunum (*r* = 0.5826, *p* < 0.0001) and Defa 21/22 in the ileum (*r* = 0.0.0302, *p* = 0.0249) (Figure 1(d)). Similarly, to the correlation with blood glucose, a consistent and strong negative correlation occurred between body fat and the expression of Defa 1-family (*r* = −0.4225, *p* = 0.0074), Defa 21/22 (*r* = −0.5128, *p* = 0.0008), CRS1C (*r* = −0.5514, *p* = 0.0003) and P-Lys (*r* = −0.4014, *p* = 0.0113), and this was specific for the ileum (Figure 1(e)). Additionally, weaker, yet significant, negative correlations were observed between Reg3g and HOMA-IR in the duodenum (*r* = −0.2868, *p* = 0.0434) and between CRS1C and body weight in the ileum (*r* = −0.2824, *p* = 0.0493) (Supplementary Figure S1d). Overall, the correlations between AMP expression and metabolic parameters were often negative, especially for blood glucose in the duodenum, and even stronger with body fat in the ileum. These findings thus suggest a defined and regio-specific response of distinct AMPs to the prolonged WSD-feeding, and that generalizing AMP response by measuring the expression of one specific AMP in one region of the small intestine only gives an incomplete representation.

The latter findings suggested that increased blood glucose or obesity during WSD consumption could in turn decrease AMP expression at these specific sites. To test whether increased blood glucose could indeed decrease AMP expression at the duodenum, we conducted an *ex-vivo* experiment where mouse duodenum and jejunum biopsies were incubated with a basolateral flow of media supplemented with glucose (Figure 1(f)). However, by using this *ex vivo* setup, which mimics the *in vivo* condition of increased glucose in circulating blood, we did not observe an effect on duodenal or jejunal Defa 21/22 or Reg3g expression when compared to the control (Figure 1(g)), implying that the increase in glucose concentration alone is not sufficient to modulate the expressions of these AMPs. Still, as the expression of Glucose transporter 2 (*Glut2*), which is expected to respond to glucose treatment, also did not significantly increase after glucose stimulation, the employed experimental conditions may not fully capture the transcriptional response of the intestinal epithelium.

Next, to investigate whether obesity itself affects AMP expression, we compared AMP expression between leptin-deficient (*Ob/Ob*^−/−^) mice and their lean wild-type littermates, both groups feeding on a chow diet (Figure 1(h)). As expected, *Ob/Ob*^−/−^ mice showed higher bodyweight (*p* = 0.0022), insulin (*p* = 0.0022) and HOMA-IR (*p* = 0.0022) levels than their lean littermates (Figure 1(i)). However, despite the severe obesity, no differences in total AMP expression (Figure 1(j)) nor in single Paneth cell AMPs (Supplementary Figure S1E-G) were observed in any small-intestinal region between the two groups. Taken together, we concluded that small intestinal AMP expression is more strongly influenced by the obesogenic diet than by obesity itself.

### Diet-dependent AMP response is influenced by mouse vendor and sex

Driven by the fact that diet was a stronger modulator of AMP expression than obesity itself, we further aimed to investigate the reason behind the conflicting findings in the literature and whether diet increases or decreases AMP expression. Accordingly, we performed different diet interventions with high-fat diet (HFD; 60% fat/20% carbohydrates) versus WSD (40% fat/40% carbohydrate), included mice from Charles River (CR) and Taconic (Tac) vendors, which are known to have different microbiota compositions,^43^ and tested both sexes (Figure 2(a-c)). After 8 weeks of intervention, AMP expression was measured to test the effect of the experimental variables diet, sex, and mouse vendor. Since most studies evaluating the impact of AMP expression have been focused on the ileum (reviewed in ref ^29^) and given that a stronger negative correlation between the diet-induced metabolic changes and AMP expression occurred in the ileum in this study (r_s_ < −0.4), we focused on investigating the changes in AMP function specifically at this site.

We first evaluated the effect of diet type (chow, WSD or HFD) on ileal AMP expression of all mice included in the different dietary interventions. While the total AMP expression, determined by the sum of the average individual AMP transcripts in each group, between chow and WSD mice was similar, the HFD-fed mice had 49% higher total AMP transcripts compared to chow-fed mice (Figure 2(d)). Similarly, the expression of individual AMPs did not yield any significant differences between mice feeding on a chow or WSD, and Pla2A2 was the only peptide which showed a significant increase under HFD-feeding relative to chow-feeding (*p* = 0.0326) (Supplementary Figure S2A). The latter suggested that a HFD had a stronger potential of influencing total AMP expression of these peptides than the WSD.

Subsequently, the effect of the variable sex on AMP expression was evaluated in the WSD dietary interventions (Figure 2(b,c)). The total AMP expression and individual AMP expression between male mice fed a chow and a WSD did not differ significantly (Figure 2(e), Supplementary Figure S2b), but WSD-female mice had a 29% lower total transcript copy number than the chow-fed females, which was mainly driven by a significantly lower expression of Reg3g (*p* = 0.0071) (Figure 2(e,f)). In addition, chow-fed females had a significantly higher Reg3g expression than chow-fed male mice (*p* = 0.0297), suggesting a sex difference in the expression of this antimicrobial protein (Figure 2(f)).

Since mouse sex significantly influenced the expression of Reg3g, we next explored whether mice vendor farm influenced AMP response under WSD-feeding in separate sexes. In male mice, we observed a significant difference in the expression of Defa21/22 (*p* = 0.0225) and P-Lys (*p* = 0.0010) between CR and Tac male mice fed the control chow diet (Supplementary Figure S2C). The latter thus suggested that baseline expression of some AMPs may already be different at the beginning of the dietary interventions, when separating vendor sources. Subsequently, we observed that WSD-feeding increased the total AMP expression by 72% in CR male mice compared to their chow-fed counterparts (Figure 2(g)), while this effect was not observed in Tac mice, thus suggesting that the mouse origin influences the response. Notably, this increase in expression was mostly driven by significantly higher levels of Reg3g transcripts in WSD-fed CR males compared to chow-fed males (*p* = 0.0498) (Figure 2(h), Supplementary Figure S2d).

Next, we wondered if the influence of the mouse vendor on total AMP expression under WSD intake would also extend to female mice. Unlike the observed differences in the expression of individual AMPs in chow-fed male mice, there was no significant difference in single AMP expression between CR and Tac female mice under chow feeding (Supplementary Figure S2E). Interestingly, while CR females showed no difference in total or single AMP expression between chow or WSD-feeding, Tac females displayed a 62% reduction in total AMP expression under WSD-feeding (Figure 2(i), Supplementary Figure S2f). Consistent with previous observations (Figure 2(f)), this reduction was mainly driven by significantly lower Reg3g expression (*p* = 0.0036, Figure 2(j)).

Taken together, compared to chow feeding, HFD-feeding had the strongest potential of influencing the total AMP expression and the individual expression of Pla2A2 when all experimental variables were combined. Furthermore, WSD-feeding only impacted total AMP expression when separating the sexes and mouse origin. Likewise, the WSD-feeding tended to increase Reg3g expression in male mice and to reduce Reg3g expression in female mice.

To complement the previous results with an additional unbiased approach, we performed a PCoA analysis to determine the effect of diet, mouse vendor, and sex on overall AMP expression (Figure 2k). This analysis revealed that all individual factors had a significant effect on the dataset (PERMANOVA diet *p* = 0.023, mouse vendor *p* = 0.001, sex *p* = 0.017), but that a combination of all factors by distinguishing the different interventions, had the strongest influence on AMP expression (*p* = 0.001; R^2^ = 0.23). This indicated that the highest variation in AMP expression occurs already during the experimental setup and thus suggests that differences in baseline expression of AMPs will have implications in the diet-mediated AMP response, which could in part explain the contrasting results between different reports in the literature.

### Ileal microbiota diversity at the mucosa or in the content was affected by mouse vendor, experiment, and the combination of diet and sex

**Figure 3.**
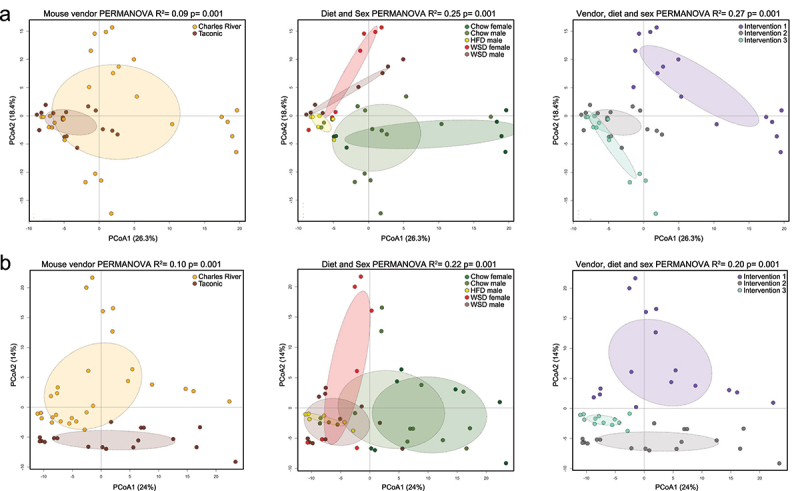
Effect of mouse vendor, combination of diet and sex and combination of vendor, diet and sex on microbiota composition in the ileal content and at the mucosa.

Intrigued by the observation that the experimental setup, defined by a combination of diet, sex, and mouse vendor, was the strongest factor modulating overall AMP expression (Figure 2(k)), we wondered if differences in microbiota composition amongst these experiments could be the underlying factor. We thus assessed the influence of mouse vendor, diet, and sex on luminal (Figure 3(a)) and mucosal (Figure 3(b)) bacterial communities in the three distinct dietary interventions (Figure 2(a–c)). To this end, we used a β-diversity analysis which quantifies differences in microbial community composition between samples, allowing to assess how distinct or similar microbiota profiles are across experimental groups. This analysis is typically visualized using ordination methods such as principal component analysis (PCoA), which cluster samples based on dissimilarity metrics, and the coefficient of determination (R2) from the PERMANOVA test can be used to indicate the variation in the distances being explained by groups under the study.

(a) Principal component analysis (PCoA) evaluating the effect of the variables mouse vendor, combination of diet and sex and combination of vendor, diet, and sex on center log ratio (CLR)-transformed genus abundance from the dietary interventions in the ileal content. (b) PCoA evaluating the effect of the variables mouse vendor, combination of diet and sex and combination of vendor, diet and sex on CLR-transformed genus abundance from the dietary interventions at the ileal mucosa. Statistical tests were calculated with a PERMANOVA analysis. * = p < 0.05, ** = p < 0.01, *** = p < 0.001, ****= < 0.0001 were considered statistically significant.

β-diversity analysis in ileal content, evaluated based on Euclidean distance, revealed clusters according to mouse vendor (PERMANOVA *p* = 0.001), the combination of diet and sex (PERMANOVA *p* = 0.001), and all factors combined (PERMANOVA *p* = 0.001) with the combination of diet and sex or all factors combined having the strongest effects (R^2^ = 0.25 and R^2^ = 0.27, respectively) (Figure 3(a)). Similarly, mouse vendor, the combination of diet and sex, and all factors combined showed significant clustering at the ileal mucosa (all *p* = 0.001), with the combination of all factors and the combination of diet and sex again having the strongest effects (R^2^ = 0.20 and R^2^ = 0.22, respectively) (Figure 3(b)). Thus, this analysis confirmed that the microbiota in the ileum content and at the mucosa differed between Charles River and Taconic vendors and showed that the combination of sex and diet had a strong effect on microbiota composition.

**Figure 4.**
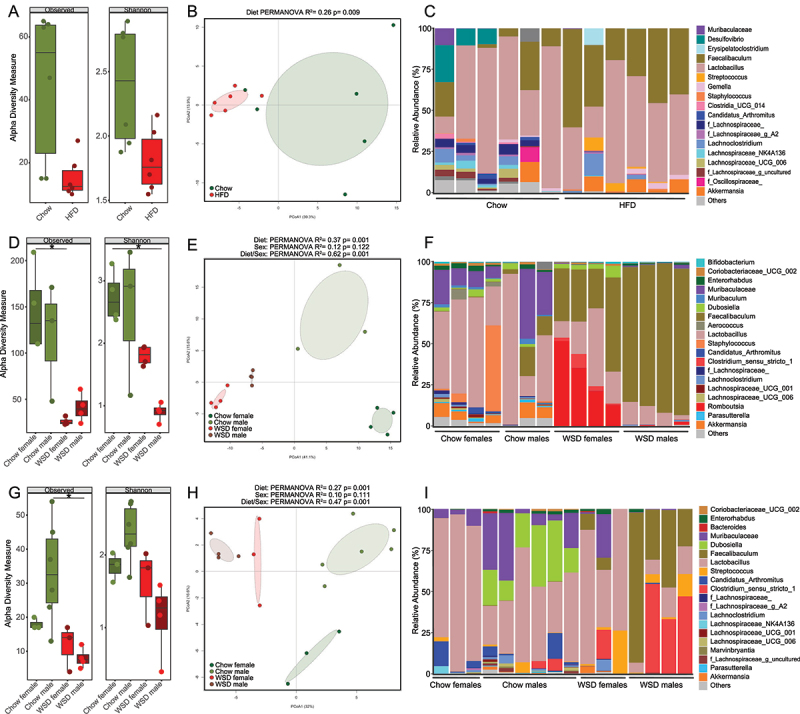
Microbiota composition in ileal content after different diet interventions.

**Figure 5.**
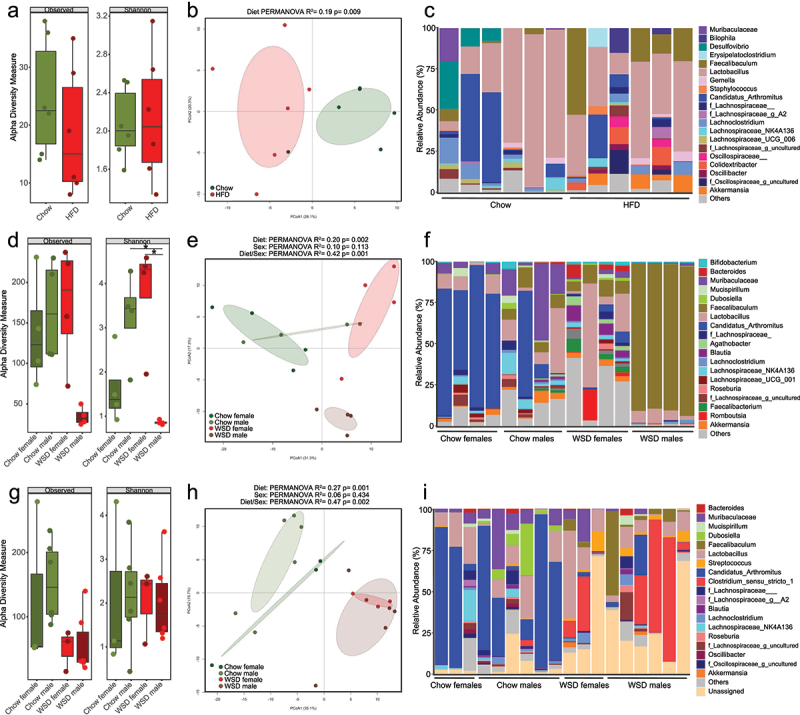
Ileal mucosa-associated microbiota composition after different diet interventions.

To next gain more insight into the specific differences in microbiota composition in the individual diet interventions (Figure 2(a-c)), we examined ileal microbiota diversity and composition in the content (Figure 4) and at the mucosa (Figure 5) for each experiment separately. Here, α-diversity was used to measure the diversity within individual microbiota samples, with observed ASV (amplicon sequence variants) which quantifies the presence of a species and with Shannon index that quantifies what is there and in which amount. As previously, β-diversity analysis was used to quantify and visualize differences in microbial community composition between samples.

(a–c) In male Charles River mice fed a chow or a high-fat diet (HFD; Figure 2(a)): (a) α-diversity according to observed ASVs and Shannon index, (b) β-diversity according to Euclidean distance between diets based on a principal component analysis (PCoA) of center log ratio (CLR)-transformed genus abundance and (c) Genera relative abundance at the ileal content. (d–f) In male and female Charles River mice fed a chow or a Western-style diet (WSD; Figure 2(b)): (d) α-diversity according to observed ASVs and Shannon index, (e) β-diversity according to Euclidean distance between diets based on a PCoA analysis of CLR-transformed genus abundance and (f) Genera relative abundance at the ileal content. (g–i) In male and female Taconic mice fed a chow or a WSD (Figure 2(c)): (g) α-diversity according to observed ASVs and Shannon index, (h) β-diversity according to Euclidean distance between diets based on a PCoA analysis of CLR-transformed genus abundance and (i) Genera relative abundance at the ileal content. Statistical differences were calculated by using Mann-Whitney U between two groups or Kruskal – Wallis for more than two groups (α-diversity), and PERMANOVA with 999 permutations (β-diversity). * = p < 0.05, ** = p < 0.01, *** = p < 0.001, ****= < 0.0001 were considered statistically significant.

(a–c) In male Charles River mice fed a chow or a high-fat diet (HFD; Figure 2(a)): (a) α-diversity according to observed ASVs and Shannon index, (b) β-diversity according to Euclidean distance between diets based on a principal component analysis (PCoA) of center log ratio (CLR)-transformed genus abundance and (c) Genera relative abundance at the ileal mucosa. (d–f) In male and female Charles River mice fed a chow or a Western-style diet (WSD; Figure 2(b)): (d) α-diversity according to observed ASVs and Shannon index, (e) β-diversity according to Euclidean distance between diets based on a PCoA analysis of CLR-transformed genus abundance and (f) Genera relative abundance at the ileal mucosa. (g–i) In male and female Taconic mice fed a chow or a WSD (Figure 2(c)): (g) α-diversity according to observed ASVs and Shannon index, (h) β-diversity according to Euclidean distance between diets based on a PCoA analysis of CLR-transformed genus abundance and (i) Genera relative abundance at the ileal mucosa. Statistical differences were calculated by using Mann-Whitney U between two groups or Kruskal – Wallis for more than two groups (α-diversity), and PERMANOVA with 999 permutations (β-diversity). * = p < 0.05, ** = p < 0.01, *** = p < 0.001, ****= < 0.0001 were considered statistically significant.

When evaluating α-diversity in the ileal content of chow- and HFD-fed male CR mice (Figure 2(a)), no statistical difference between the two groups was observed, likely due to the variability in the chow group (Figure 4(a)). Likewise, despite that chow and HFD-fed mice clustered separately according to β-diversity analysis using a PCoA based on Euclidean distance (R^2^ = 0.26, *p* = 0.009 Figure 4(b)), the chow-fed mice displayed high inter-group variability. On genus level, increased relative abundance of *Faecalibaculum* and *Akkermansia* were observed in the HFD-fed mice when compared to the chow-fed group (Figure 4(c) Supplementary Figure S3a). In addition, HFD feeding decreased the abundance of *Lachnospiraceae_UCG-006* (Figure 4(c), Supplementary Figure S3a).

We next evaluated the effect of WSD-feeding in CR male and female mice (Figure 2(b)). Consistent with previous observations under HFD-feeding, male CR mice feeding on a WSD had similar α-diversity when compared to chow-fed mice (Figure 4(d)). However, female mice on a WSD diet had a strong reduction in Observed ASVs (*p* < 0.05), but not in Shannon index, when compared to chow-fed CR females (Figure 4(d)). Again, diet showed a significant separation according to a PCoA based analysis (R^2^ = 0.37 *p* = 0.001), which was even more evident when combining the effect of diet and sex (R^2^ = 0.62, *p* = 0.001 Figure 4(e)). On genus level, *Faecalibaculum* had a higher abundance in chow-fed males than chow-fed females and was significantly increased under WSD-feeding compared to chow feeding in both sexes (Figure 4(f), Supplementary Figure S3b). Similarly, *Romboutsia* was increased under WSD-feeding in both sexes, and WSD-fed females mice exhibited higher abundance than the males. *Candidatus arthromitus*, which was observed in male and female mice fed a chow diet, was absent under WSD-feeding in both sexes. Likewise, the abundance of *Muribaculaceae* decreased under WSD-feeding compared to chow feeding (Figure 4(f), Supplementary Figure S3b). Furthermore, WSD fed male and female mice had decreased relative abundance of *Akkermansia* and *Parasutterella* than chow-fed mice. Interestingly, the decrease in relative abundance of *Akkermansia* observed in this experiment contrasts the observations from the HFD intervention (Supplementary Figure S3A), and suggests that this bacterium can react differently to a WSD or HFD in the ileal content (Figure 4(f), Supplementary Figure S3b). Overall, diet was thus the main factor explaining the variation in the relative abundance of the altered taxa (94% *Parasutterella*, 78% *Akkermansia*, 72% *Faecalibaculum*, 68% *Romboutsia*, 53% *Candidatus arthromitus*, 33% *Muribaculaceae*), and thus highlights them as highly sensitive to dietary modulation. However, smaller yet significant differences were also observed based on sex (2-way ANOVA variation driven by sex: 12% *Faecalibaculum* and 11% *Romboutsia*).

Next, when comparing the effect of WSD-feeding in a different mouse vendor (Figure 2(c)), the Tac male mice fed a WSD had a strong reduction in Observed ASVs (*p* < 0.05), but not in Shannon index, when compared to chow-fed males (Figure 4(g)). In contrast, no significant difference was observed between chow- and WSD-fed Tac females (Figure 4(g)). Consistent with previous observations, diet caused a significant clustering according to Euclidean distance (R^2^ = 0.27, *p* = 0.001), and this effect was even stronger when diet and sex were combined (R^2^ = 0.47, *p* = 0.001 Figure 4(h)). In accordance with previous findings, there was an enrichment of *Faecalibaculum* and a reduction of *Muribaculaceae* in the ileal content of male Tac mice fed a WSD relative to chow-fed males, and WSD fed females had a lower abundance of *Candidatus arthromitus* when compared to chow-fed females. Also consistent with previous findings, diet had the strongest influence on the ileal luminal abundance of these genera in Tac mice (2-way ANOVA variation driven by diet: 61% *Faecalibaculum*, 51% *Muribaculaceae*, 50% *Candidatus arthromitus*).

Collectively, this analysis revealed variations in the α-diversity of the ileal luminal microbiota in response to high-fat diet feeding amongst mice groups across experiments. Moreover, significant clustering was observed in all experiments based on β-diversity analysis, mainly reflecting distinctions based on diet and, to an even greater extent, on the combination of diet and sex.

### Differences in ileal microbiota composition at the mucosa

While luminal microbiota may affect AMP expression through secretion of metabolites, mucosal microbiota may have a direct contact with the host mucosa. We thus analyzed the mucosa-associated microbiota at the ileum from the different diet interventions (Figure 2(a–c)). When assessing alterations in α-diversity at the ileal mucosa of male CR mice fed either chow or a HFD (Figure 2(a)), no significant difference was observed between the groups (Figure 5(a)). Yet, diet led to distinct clusters in the β-diversity PCoA analysis (R^2^ = 0.19, *p* = 0.009 Figure 5(b)). Changes in genus abundance in the ileal mucosa were characterized by an increase in abundance of *Akkermansia* and decrease of *Lachnospiraceae_UCG-006* under HFD feeding compared to chow feeding (Figure 5(c), Supplementary Figure S4a), supporting the observations made in the ileal content of these mice. However, although there was a trend toward increased *Faecalibaculum* abundance in the mucosa of HFD fed mice, this did not reach statistical significance.

When examining the impact of a WSD compared to a chow diet on the ileal mucosa in both male and female CR mice (Figure 2(b)), WSD-fed male mice had a significant decrease in Shannon index (*p* < 0.05) (Figure 5(d)). In accordance with previous observations, a β-diversity PCoA analysis showed a significant separation according to diet (R^2^ = 0.20, *p* = 0.002), with a clearer separation when diet and sex were combined (R^2^ = 0.42, *p* = 0.001 Figure 5(e)). Consistent with findings on the ileal content, *Faecalibaculum* abundance was higher in male mice when compared to females, independent of diet, and the abundance of this microbe was significantly increased in both sexes after WSD-feeding (Figure 5(f), Supplementary Figure S4b). Interestingly, the abundance of *Candidatus arthromitus* was significantly lower in the ileal mucosa of chow males compared to chow females, and the abundance of both *Candidatus arthromitus* and *Muribaculaceae* decreased significantly in both sexes after WSD-feeding. In contrast, the abundance of *Blautia* was increased in female mice feeding on WSD compared to chow diet. Furthermore, WSD-fed male mice had significantly lower levels of *Akkermansia* and *Dubosiella* when compared to the other three mouse groups. The susceptibility of these genera toward diet was also confirmed (2-way ANOVA variation driven by diet: 56% *Muribaculaceae*, 52% *Akkermansia*, 39% *Candidatus arthromitus*, 36% *Faecalibaculum*, 19% *Dubosiella*). However, notable differences were also observed according to sex (2-way ANOVA variation driven by sex: 45% *Faecalibaculum*, 29% *Candidatus arthromitus*, 23% *Akkermansia*).

When analyzing the ileal mucosa microbiota in male and female mice obtained from Taconic vendors (Figure 2(c)), WSD-feeding did not significantly affect α-diversity (Figure 5(g)). Yet, β-diversity analysis revealed a significant clustering according to diet (R^2^ = 0.27, *p* = 0.001), which was more significant when combining diet and sex (R^2^ = 0.47, *p* = 0.002 Figure 5(h)). When compared to chow feeding, the mucosal microbiota of male mice feeding on a WSD was characterized by a decrease in *Candidatus arthromitus* and *Muribaculaceae* (Figure 5(i), Supplementary Figure S4c). Like in the content of these mice, the intake of a WSD also increased *Faecalibaculum* abundance in both sexes relative to chow. In addition, *Dubosiella* abundance was significantly higher in chow-fed males compared to the other mouse groups. Consistent with previous observations, diet had the strongest impact on the abundance of most of these taxa (2-way ANOVA variation driven by diet: 53% *Faecalibaculum*, 52%, *Candidatus arthromitus*, 26% *Muribaculaceae*), and *Dubosiella* was significantly driven by sex and the interaction between diet and sex (2-way ANOVA sex: 29% and interaction between diet and sex: 30%).

In summary, the luminal and mucosal microbiota composition of all experiments was distinct and showed different responses to dietary challenge. However, despite this, a few consistent shifts in relative abundance were observed between the studies, including an increase in *Faecalibaculum* and a reduction in *Muribaculaceae* and *Candidatus arthromitus* under WSD-feeding. Additionally, while α- and β-diversity metrics showed greater variation in chow-fed mice, there was improved clustering observed under WSD or HFD feeding, which was more evident in the luminal microbiota. This suggests that the luminal microbiota is more responsive to dietary challenges compared to the mucosal microbiota.

### Correlation analysis revealed different interactions between AMP expression and small intestinal bacterial abundance at different sites

As we identified the combination of various experimental factors as the strongest variable influencing both AMP and microbiota composition, it is possible that the different microbiota configurations in the experiments influenced the previously observed differences in individual AMP expression following WSD or HFD feeding. To test this, we used a Procrustes analysis to assess the concordance between microbiome profiles and AMP expression across all samples together and within the sub-groups of chow diet, WSD and HFD, and further validated this association with a Mantel test. When considering all samples together in both content and mucosa samples, the overall concordance between microbiome and AMP data was moderate (Supplementary Figure S5A: content m12^2^ = 0.822, *p* = 0.001; Supplementary Figure S5B MAB m12Â^2^ = 0.851 *p* = 0.003) and more prominent during WSD feeding (Supplementary Figure S5C: content m12^2^ = 0.522, *p* = 0.003; Supplementary Figure S5D MAB m12Â^2^ = 0.622 *p* = 0.007) than during chow or HFD feeding. This suggests that the link between microbial composition and AMP expression is present across diets, but most pronounced under WSD conditions.

We next aimed to identify potential AMP-microbiota interactions in our experiments and investigated whether the bacteria in the small intestinal content or at the mucosa would correlate with AMP transcript copy numbers. For this purpose, we performed a correlation analysis using HAllA^41^ between individual AMP expression and bacterial abundances in the content and mucosa of the mice fed chow, WSD, HFD or all diets combined (Figures 2(a-c)). When examining the interactions in the content in all diet interventions combined, a positive correlation was observed between *Bifidobacterium* and CRS1C (*r* = 0.5342, p_adj_ = 0.0484), *Staphylococcus* and Reg3g (*r* = 0.5909, p_adj_ = 0.0127) and Pla2A2 (*r* = 0.5840, p_adj_ = 0.0127) (Supplementary Table S1). In contrast, significant negative associations were observed at the mucosa: Reg3g correlated negatively with *Candidatus_Azambacteria* (*r* = −0.4857, p_adj_ = 0.0522) and *Enterococcus* (*r* = −0.5073, p_adj_ = 0.0369), Reg3g and pLysosyme both correlated negatively with *Clostridium_sensu_stricto_1* (*r* = −0.5768 p_adj_ = 0.0106 and *r* = −0.5342 p_adj_ = 0.0215), *Streptococcus* (*r* = −0.5755 p_adj_ = 0.0106 and *r* = −0.5703 p_adj_ = 0.0106) or an unassigned taxon (*r* = −0.6702 p_adj_ = 0.0005 and *r* = −0.6240 p_adj_ = 0.0028) while all tested AMPs correlated negatively with *Pseudomonas* (*r* < −0,4926, p_adj_ < 0,04838) (Supplementary Table S1).

**Figure 6.**
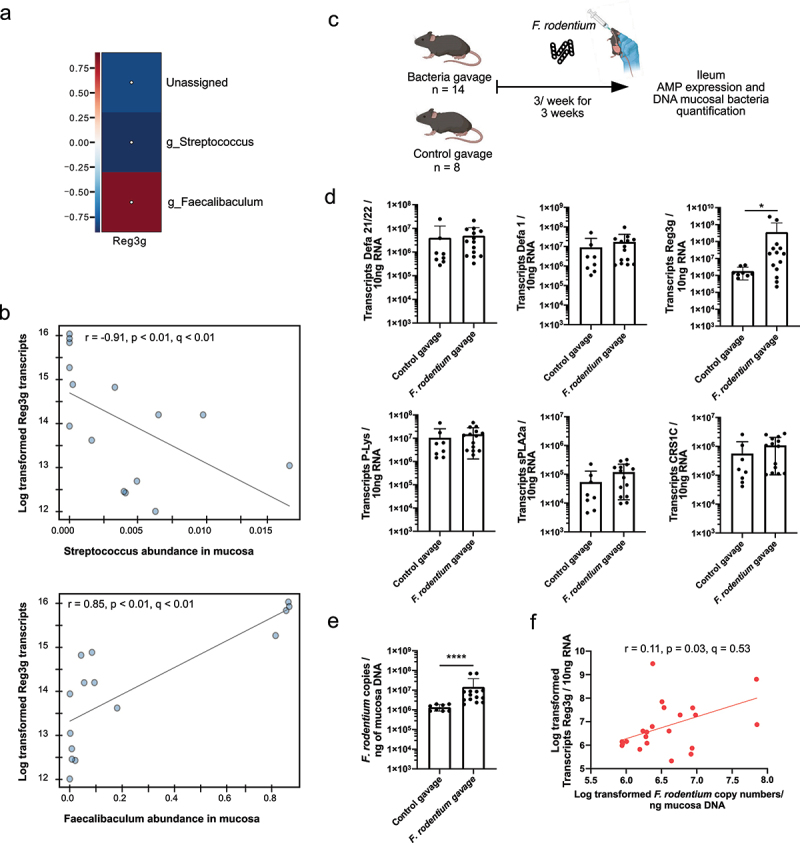
Correlations between AMP expression and mucosal small intestinal microbiota revealed a novel microbe capable of inducing Reg3g expression.

When testing chow diet or HFD feeding individually (Supplementary Table S1), we did not identify any significant correlation between individual bacterial abundance in the content or at the mucosa and AMP expression. However, in mice feeding on a WSD, we observed that Reg3g had a negative correlation with *Streptococcus* abundance in the content (*r* = −0.8154 p_adj_ = 0.0456) and at the mucosa (*r* = −0.9063 p_adj_ = 0.0004) (Figure 6(a,b), Supplementary Table S1). Furthermore, the strongest positive correlation of all analyses was observed between the abundance of *Faecalibaculum* at the mucosa and Reg3g (*r* = 0.8480, p_adj_ = 0.0067) (Figure 6(a,b), Supplementary Table S1). Curiously, the abundance of this microbe was significantly boosted during WSD-feeding (Supplementary Figures S3B,C and 4(b,c)) and was consistently modified according to diet based on a 2-way ANOVA analysis. We thus hypothesized that this increased abundance in response to the WSD intake could in turn increase Reg3g expression. To test this hypothesis, we repeatedly gavaged mice fed a chow diet with *Faecalibaculum rodentium* and analyzed AMP expression after 3 weeks (Figure 6(c)). And indeed, supplementation of *F. rodentium* specifically increased the expression of Reg3g, but not of other AMPs, including Defa21/22, Defa1-family, lysozyme, CRS1C or sPLA2a in the ileum (Figure 6(d)). Moreover, the mucosal abundance of *F. rodentium* increased in the mice gavaged with this bacterium, demonstrating that this microbe can also adhere to the mucosal compartment when supplemented orally (Figure 6(e)). As the increase in Reg3g expression also correlated with the *F. rodentium* copy numbers measured in the ileal mucosa of these mice (Figure 6(f)), we conclude that this microbe is capable of specifically inducing Reg3g expression in the mouse ileum.

(a) HAIIA representation of spearman correlations between log transformed AMP transcripts and relative abundance of microbiota in WSD only from the dietary interventions in in mucosa. (b) Representative scatterplots of significant correlations identified by HAllA, between Reg3g transcripts and *Streptoccocus* and *Faecalibaculum* mucosal abundance in WSD fed mice. (c) Schematic experimental set-up of mice fed a chow diet and gavaged with a *Faecalibaculum rodentium* suspension or reduced PBS (control treatment) 3 times per week for 3 weeks. (d) Absolute expression of Defa1, Defa21/22, P-Lysozyme (P-Lys), CRS1C, Reg3g and sPLA2a in the ileum of the mice from the *F. rodentium* gavage experiment. (e) *F. rodentium* copy numbers measured in the ileal mucosa of the *F. rodentium* gavage experiment. (f) Spearman correlation between Reg3g transcript copy numbers and log transformed *F. rodentium* copy numbers in mucosa DNA measured in the ileum of the mice in the *F. rodentium* gavage experiment. Mann-Whitney U test was used to determine statistical significance in panels D and E. * = p < 0.05, ** = p < 0.01, *** = p < 0.001, ****= < 0.0001 were considered statistically significant. Statistically significant correlations in panels B and F are indicated by colored lines and correlation coefficients with *p*- and q-values are shown.

In summary, this analysis revealed strong and specific correlations between distinct AMPs and bacterial taxa, particularly under WSD-feeding. Notably, the commensal bacterium *F. rodentium* was significantly enriched by WSD-feeding and we confirmed its ability to directly induce Reg3g expression, suggesting a causal link. These findings thus identify a novel diet-sensitive host–microbe interaction that may be exploited to beneficially modulate AMP expression and support intestinal health.

## Discussion

The human intestine is at the center of the intricate interaction between gut bacteria and AMPs. While the presence of the microbiota can stimulate AMP expression, AMPs play a crucial role in maintaining a safe distance between trillions of microbes and the epithelial mucosa. However, our understanding of how dietary patterns influence this interaction, especially within the small intestine, remains relatively limited.

In this study, we evaluated the impact of different high-fat diets and obesity on AMP expression in mice. We found that obesity-inducing WSD, but not the obesity phenotype per-se, moderately modulated AMP expression. The latter has a potential translational implication as it suggests that obese individuals eating a healthy-balanced diet may have an adequate AMP response when compared to obese individuals consuming a caloric WSD; yet this remains to be studied in human cohort studies.

Notably, when evaluating the duration of WSD feeding on AMP expression, changes in AMP expression following a long-term consumption of a WSD correlated negatively with systemic blood glucose in the duodenum and with body fat in the ileum, indicating a regio-specific connection between diet-induced metabolic dysfunction and intestinal AMPs. As hyperglycemia has been previously associated with intestinal epithelial cell transcriptional reprogramming and impaired host intestinal barrier,^44^ it is also possible that the WSD negatively impacts AMP function at specific sites of the small intestine. Interestingly, the observed negative correlations are in line with our previous study, in which defensin-deficient mice had a more pronounced increase in blood glucose levels or body fat upon WSD-feeding when compared to WT mice,^7^ suggesting an inverse association between blood glucose and/or body fat and AMP expression. However, as we show here (Figure 1(f,g)), increased glucose levels were not sufficient to reduce AMP expression. These findings are supported by a previous study in mice, which investigated the effect of hyperglycemia on epithelial barrier dysfunction.^44^ In that study, host defense gene expression, including Reg3g remained unaltered between a control treatment and hyperglycemic mice. Of note, our *ex vivo* glucose stimulation experiment has been performed under a short one-hour period due to the viability of *ex vivo* biopsies. It thus remains unclear whether higher glucose concentrations or longer incubation periods would have led to a glucose-dependent response. Nevertheless, in agreement with previous literature, our findings suggest that blood glucose alone cannot modulate AMP expression, and that additional factors occurring during metabolic dysfunction may play a role in this association.

Intake of WSD is associated with an increased risk of chronic inflammation and metabolic diseases such as obesity and type 2 diabetes.^22,45^ However, only a few studies have investigated metabolic dysfunction in the context of the antimicrobial response at the intestinal mucosa. Initial evidence shown in obese patients revealed a negative correlation between jejunal lysozyme protein levels and body mass index (BMI).^21^ Later, Larsen *et al*. described that HFD oral supplementation with HD5 protected mice from an increase in circulating levels of cholesterol, fatty acids, and insulin resistance, without influencing body weight gain.^32^ Similarly, Shin *et al*. discovered that the effect of vertical sleeve gastrectomy, a procedure meant to induce weight loss, and the administration of the fermentable fiber inulin, exerted a beneficial effect on preventing glucose dysfunction during diet-induced obesity, and that this effect was dependent on the presence of *Reg3g*^17^. The beneficial effect of Reg3g was further confirmed by peritoneal injection of this protein prior to an oral glucose tolerance test, which resulted in a mild reduction in blood glucose levels in mice.^17^ Therefore, several lines of research show that AMP supplementation has the potential to ameliorate metabolic dysfunction during diet-induced obesity, yet the mechanisms behind this effect are not completely understood.

Independent of diet, leptin-deficient *Ob/Ob*^−/−^ mice have been shown to have significantly lower expression of *Reg3g* in the ileum but not in the duodenum or jejunum when compared to WT mice.^17^ In contrast to these findings, we did not observe any significant changes in ileal *Reg3g* transcript levels between *Ob/Ob*^−/−^ and their lean littermates. It is possible that the difference is caused by details in the experimental setup. Here, we used tightly controlled littermates that were separated based on genotype directly after weaning, and all mice were bred and maintained in the same environment.

Mice from different vendors have different microbiota composition (Figure 3(a,b)), and the environment in different animal facilities will also affect the local mouse microbiota. Interestingly, we observed some differences in AMP expression between mice from different vendor sources, and when comparing ileal AMP expression levels of our chow-fed mice to the few other studies using absolute transcript quantification,^14,31^ we observed 10–100 times higher baseline expression of *Reg3g* in our mouse colony, indicating that the difference in baseline expression of *Reg3g* between animal facilities is far higher than the reported diet-induced changes. Of note, when comparing expression levels of Defa21/22 expression (previously described as Crypt4), we observed similar expression levels between our study and published studies,^14,31^ with ca. 5 × 10^6^ transcripts/10 ng RNA from ileum biopsies in C57BL/6 mice. Thus, these findings allow two important conclusions: first, the methodology to measure absolute expression transcripts is consistent and comparable across different studies. Second, α-defensin expression is constitutive once a baseline microbiota is present, and specific microbes have a rather minor influence. This is also in line with the findings of this study where α-defensin expression was not significantly changed upon HFD- or WSD-feeding, despite that both diets induced changes in the small intestinal microbiota composition in the content and at the mucosa.

Our study aimed to gain deeper insights into the discrepancies found in the literature regarding the effects of high-fat diet intake on small intestinal AMP expression. While some studies indicate a reduced expression of specific AMPs under WSD or HFD feeding,^17,23–26^ others have reported an increase in AMP expression.^27,28^ Of note, the WSD-induced reduction in the expression of *Reg3g* was not consistent across different regions of the small intestine in these studies, further supporting that the expression of this protein is highly variable not only between animal facilities but also along the small intestine. In addition, most of these studies performed a relative abundance expression analysis and compared the diet intervention to a chow-fed control group. While this method has the advantage of comparing the influence of a specific treatment with a control condition, it does not consider the potential differences in its baseline expression and is highly sensitive to variations in the choice of reference genes for normalization.

In this investigation, and confirming our previous findings,^7^ we observed that the absolute transcript numbers of individual ileal AMPs remained largely unchanged between chow and WSD-fed mice in three distinct mouse experiments. While this may appear unexpected, our study provides a potential explanation by identifying that experimental variables, including mouse vendor, sex, and diet type, have a strong influence on ileal AMP expression, potentially by affecting the gut microbiota composition.

Another factor that may affect the results from high-fat diet intervention studies is the variation in their duration. However, as we discussed previously,^29^ studies report both increased and decreased AMP expression under HFD compared to a control diet, with no consistent link to feeding duration. In this study, when evaluating the duration of WSD feeding on AMP expression (Figure 1(a)), we observed regio-specific changes in AMP expression over time, although the absence of a control diet group limits a direct comparison.

As an example of the impact of the different variables on individual AMP expression, when combining all mice, we observed that *Pla2A2* was the only AMP that showed a significant increase in HFD fed mice relative to chow-fed mice. In addition, *Reg3g* exhibited a significantly lower expression in female Taconic mice when compared to chow-fed mice. In contrast, male mice obtained from Charles River had significantly higher *Reg3g* expression after WSD-feeding when compared to chow feeding. Therefore, while *Reg3g* was the antimicrobial protein that was most influenced by high-fat diets, the response differed between sexes and origin of the mice.

Previous research has shown that expression of the AMP *Reg3g* can be induced by probiotic supplementation with *Akkermansia muciniphila* or *Bacteroides thetaiotaomicron* in conventional mice^16,30^ or with *Bifidobacterium breve* in GF mice.^46^ In addition, conventionalized GF mice also had increased expression of several AMPs,^14,30,46^ suggesting that the presence of the microbiota and possibly, of specific bacteria, has the potential to modulate AMP expression. When exploring this possibility in this study, we found that the relative abundance of several bacteria in the small intestine correlated with specific individual AMPs, and these correlations were influenced by both diet and the location within the intestinal compartments. When evaluating the associations between AMPs and small intestinal bacteria abundance, we observed a significant positive correlation between the mucosa abundance of *Faecalibaculum* and *Reg3g* expression under WSD-feeding. Specifically, *Faecalibaculum* was significantly enriched by WSD or HFD intake in our study, which was also previously observed by us in the small intestine^3,7^ and by others in the colon.^47^ Interestingly, *Faecalibaculum* growth was shown to be promoted by sucrose or maltodextrin commonly present in WSDs,^47^ and this finding provides an explanation for the enrichment of *Faecalibaculum* in the microbiota of WSD-fed mice. This finding was corroborated by oral supplementation of *F. rodentium* to chow-fed mice, which increased *Reg3g* expression in the ileum. This confirmed that this microbe can induce *Reg3g* expression even in the absence of a WSD. *F. rodentium* is a Gram-positive and strict anaerobe which is able to ferment glucose into lactic acid.^48^ In turn, lactic acid was found to increase the expression of *Reg3g* in intestinal enteroids,^17^ providing a possible explanation for the *F. rodentium*-mediated *Reg3g* induction in small intestinal mucosa.

In conclusion, this study reveals a regio-specific association between antimicrobial peptide expression and key metabolic parameters. Nevertheless, the diet-independent obesity model and our *ex-vivo* glucose stimulation experiment both indicate – despite their limitations – that there is no direct effect of obesity or increased glucose levels on AMP expression and suggest that intake of the obesogenic WSD has a stronger potential to modulate AMP expression. However, the association between metabolic parameters and AMP expression could also be an indirect correlation caused by diet-mediated changes on the microbiome. Furthermore, *Reg3g* was the antimicrobial protein that was most responsive to high-fat diets, although the response differed between sexes and origin of the mice, and was additionally inducible by a specific gut microbe present at the mucosa. Therefore, novel therapeutic strategies aiming to correct the dysregulation of AMP expression during disease should consider that not all AMPs may be modifiable by dietary intervention or distinct bacteria. Lastly, the strong influence of experimental variables – such as mouse vendor, sex, and diet type – on AMP expression may explain discrepancies in previous studies and highlights the need for careful experimental design and interpretation when assessing the impact of high-fat diets on the intestinal antimicrobial response.

## Supplementary Material

Supplemental Material

Supplementary Figures.pdf
