# Supplementary material for: Disentangling the impact of obesity, diet, host factors, and microbiota on small intestinal antimicrobial peptide expression
Source: Gut Microbes. 2025 Aug 4;17(1):2536095. doi: 10.1080/19490976.2025.2536095 (PMC12326570; doi:10.1080/19490976.2025.2536095)
Supplement: Supplemental Material [file KGMI_A_2536095_SM3427.docx]

## **Supplementary Figures**

**Supplementary Figure 1. Single AMP expression in diet induced obesity and genetic obesity**

(A-C) Expression of Paneth-cell AMPs of the WSD duration experiment: Defa1, Defa21/22, P-Lysozyme (P-Lys), CRS1C, Reg3g and sPLA2a at the (A) duodenum; (B) jejunum and (C) ileum. (D) Statistically significant correlations between individual AMP expression at the duodenum (red), jejunum (blue) and ileum (black) of the WSD duration experiment with HOMA-IR and bodyweight. Pearson (normally distributed data) or Spearman (not-normally distributed data) correlation analysis was used to test correlations between AMP expression and metabolic parameters, and only the statistically significant correlation coefficients with p-and -q-values are shown. (E-G) Expression of Paneth-cell AMPs of the genetic obesity experiment: Defa1, Defa21/22, P-Lys, CRS1C, Reg3g and sPLA2a at the (E) duodenum; (F) jejunum and (G) ileum. Statistical tests were calculated with a Mann-Whitney U test for the individual AMP expression analysis. *= p< 0.05, **= p< 0.01, ***= p< 0.001, ****= <0.0001 were considered statistically significant.

**Supplementary Figure 2. AMP expression under chow-diet feeding and in response to dietary intervention in male or female mice from different vendors**

(A) Expression of Paneth-cell AMPs Defa1, Defa21/22, P-Lysozyme (P-Lys), CRS1C, Reg3g and sPLA2a in the ileum of all mice from the dietary interventions fed a chow, WSD or HFD (Figure 2A, B, C). (B) Expression of Paneth-cell AMPs in the ileum of male and female mice fed a chow or WSD (Figure 2B, C). (C) Expression of Paneth-cell AMPs in the ileum of male mice from Charles River (Figure 2A, B) fed a chow diet. (D) Paneth-cell AMP transcript copy number in the ileum of male mice from CR (Figure 2B) or Tac (Figure 2C) fed a chow or a WSD. (E) Expression of Paneth-cell AMPs in the ileum of female mice from CR (Figure 2B) and Tac (Figure 2C) fed a chow diet. (F) Paneth-cell AMP transcript copy number in the ileum of female mice from CR (Figure 2B) or Tac (Figure 2C) fed a chow or a WSD. Statistical tests were calculated with a One-way with Dunn’s multiple comparison test between WSD fed groups compared to the chow control group (A, D, F), with a 2-Way ANOVA with Tukey’s Multiple comparison for AMP quantification between sexes (B), and with a Mann-Whitney U test when comparing AMP expression between two groups (C, E). *= p< 0.05, **= p< 0.01, ***= p< 0.001, ****= <0.0001 were considered statistically significant.

**Supplementary Figure 3. Differentially abundant genera at the ileal content.**

(A) Center log ratio (CLR)-transformed genera in the ileum content of male Charles River mice fed a chow or a high-fat diet (HFD; Figure 2A). (B) CLR-transformed genera in the ileum content of male and female Charles River mice fed a chow or a Western-style diet (WSD; Figure 2B). (C) CLR-transformed genera in the ileum content of male and female Taconic mice fed a chow or a WSD (Figure 2C). Statistical tests were calculated with a Mann-Whitney U test when comparing AMP expression between two groups and with a 2-way ANOVA with Tukey’s multiple comparisons test when comparing more than two groups. *= p< 0.05, **= p< 0.01, ***= p< 0.001, ****= <0.0001 were considered statistically significant.

**Supplementary Figure 4. Differentially abundant genera at the ileal mucosa-associated microbiota**

(A) Center log ratio (CLR)-transformed genera in the ileum mucosa of male Charles River mice fed a chow or a high-fat diet (HFD; Figure 2A). (B) CLR-transformed genera in the ileum mucosa of male and female Charles River mice fed a chow or a Western-style diet (WSD; Figure 2B). (C) CLR-transformed genera in the ileum mucosa of male and female Taconic mice fed a chow or a WSD (Figure 2C). Statistical tests were calculated with a Mann-Whitney U test when comparing AMP expression between two groups and with a 2-way ANOVA with Tukey’s multiple comparisons test when comparing more than two groups. *= p< 0.05, **= p< 0.01, ***= p< 0.001, ****= <0.0001 were considered statistically significant.

**Supplementary Figure 5. Procrustes and Mantel analyses reveal concordance between microbiota composition and antimicrobial peptide (AMP) expression across diets.**

(A - D) Procrustes analysis performed to assess the association between microbiota relative abundance and AMP transcript expression across all diets (chow, WSD, HFD) in small intestinal content (A) and mucosa samples (B) and in WSD diet subgroup in content (C) and in mucosa (D). The congruence between datasets was quantified using the sum of squared distances between matched sample points after optimal superimposition (m12²), with statistical significance assessed via permutation (n = 999) testing. A Mantel test was used to validate these associations.

**Supplementary Table 1.** All HAIIA significant associations between relative genus abundance and log-transformed AMP transcripts in all diets, chow, Western-style diet and High-fat diet in content or mucosa after multiple correction (q-val). Related to Figure 6.

| **Content all diets together** | | | | |
| --- | --- | --- | --- | --- |
| **Bacteria genus** | **AMP** | **association** | **p-values** | **q-values** |
| g__Bifidobacterium | CRS1C | 0,534241 | 0,000224 | 0,048446 |
| g__Staphylococcus | Reg3g | 0,590986 | 3,01E-05 | 0,01271 |
| g__Staphylococcus | Pla2A2 | 0,584092 | 3,92E-05 | 0,01271 |
| **Mucosa all diets together** | | | | |
| **Bacteria genus** | **AMP** | **association** | **p-values** | **q-values** |
| g__Candidatus_Azambacteria | Reg3g | -0,48572 | 0,000719 | 0,05226 |
| g__Pseudomonas | Defa1 | -0,52265 | 0,00023 | 0,025892 |
| g__Pseudomonas | Defa21-22 | -0,50618 | 0,000389 | 0,036977 |
| g__Pseudomonas | Reg3g | -0,52682 | 0,000201 | 0,024846 |
| g__Pseudomonas | CRS1C | -0,4926 | 0,000587 | 0,04838 |
| g__Pseudomonas | pLysozyme | -0,55958 | 6,43E-05 | 0,012782 |
| g__Pseudomonas | Pla2A2 | -0,50274 | 0,000432 | 0,038174 |
| g__Enterococcus | Reg3g | -0,50732 | 0,000375 | 0,036977 |
| g__Clostridium_sensu_stricto_1 | Reg3g | -0,57682 | 3,36E-05 | 0,010662 |
| g__Clostridium_sensu_stricto_1 | pLysozyme | -0,53427 | 0,000157 | 0,021521 |
| g__Streptococcus | Reg3g | -0,57552 | 3,53E-05 | 0,010662 |
| g__Streptococcus | pLysozyme | -0,57032 | 4,31E-05 | 0,010662 |
| Unassigned | Reg3g | -0,6702 | 4,76E-07 | 0,000588 |
| Unassigned | pLysozyme | -0,62408 | 4,63E-06 | 0,002864 |
| **Content chow diet** | | | | |
| No significant association | | | | |
| **Mucosa chow diet** | | | | |
| No significant association | | | | |
| **Content Western-style diet** | | | | |
| **Bacteria genus** | **AMP** | **association** | **p-values** | **q-values** |
| g__Streptococcus | Reg3g | -0,81543 | 0,000211 | 0,045657 |
| **Mucosa Western-style diet** | | | | |
| **Bacteria genus** | **AMP** | **association** | **p-values** | **q-values** |
| Unassigned | Reg3g | -0,82292 | 4,98E-05 | 0,013136 |
| g__Streptococcus | Reg3g | -0,90631 | 5,41E-07 | 0,000429 |
| g__Faecalibaculum | Reg3g | 0,848039 | 1,71E-05 | 0,006757 |
| **Content High-fat diet** | | | | |
| No significant association | | | | |
| **Mucosa High-fat diet** | | | | |
| No significant association | | | | |
