# Supplementary material for: Disentangling the impact of obesity, diet, host factors, and microbiota on small intestinal antimicrobial peptide expression
Source: Gut Microbes. 2025 Aug 4;17(1):2536095. doi: 10.1080/19490976.2025.2536095 (PMC12326570; doi:10.1080/19490976.2025.2536095)
Supplement: Supplementary Figures.pdf [file KGMI_A_2536095_SM5761.pdf]

# Supplementary Figure 1

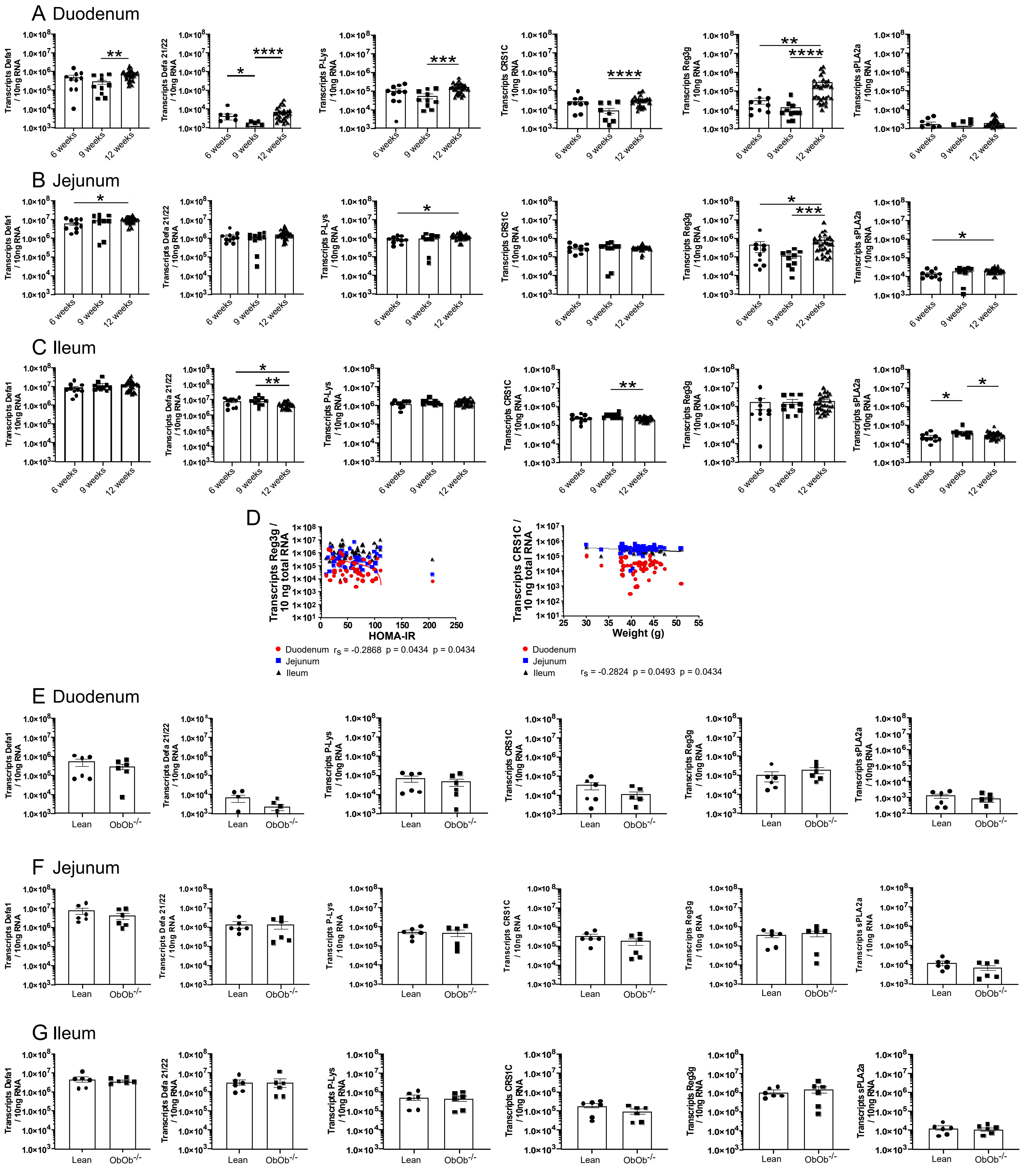

Supplementary Figure 2

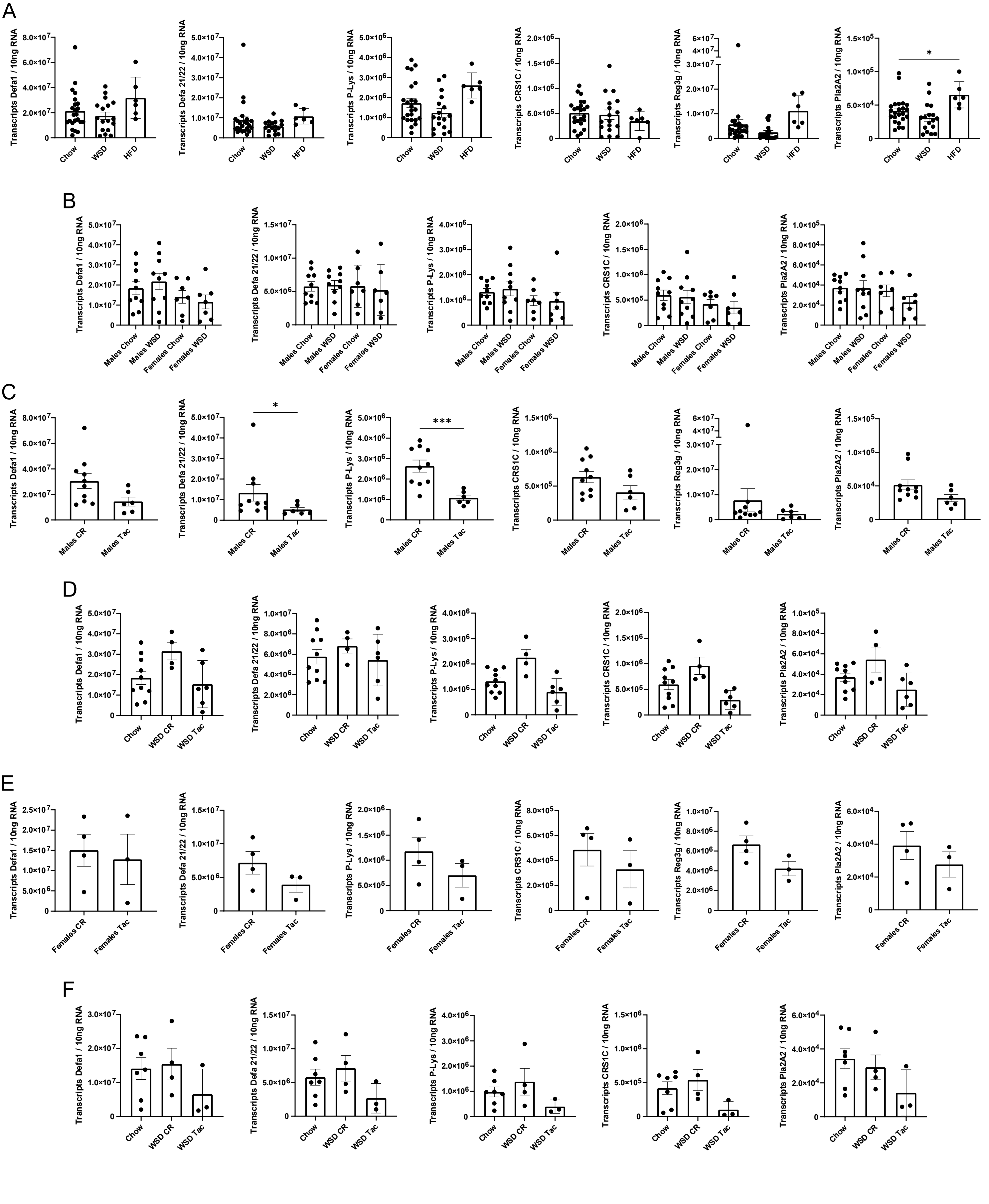

# Supplementary Figure 3

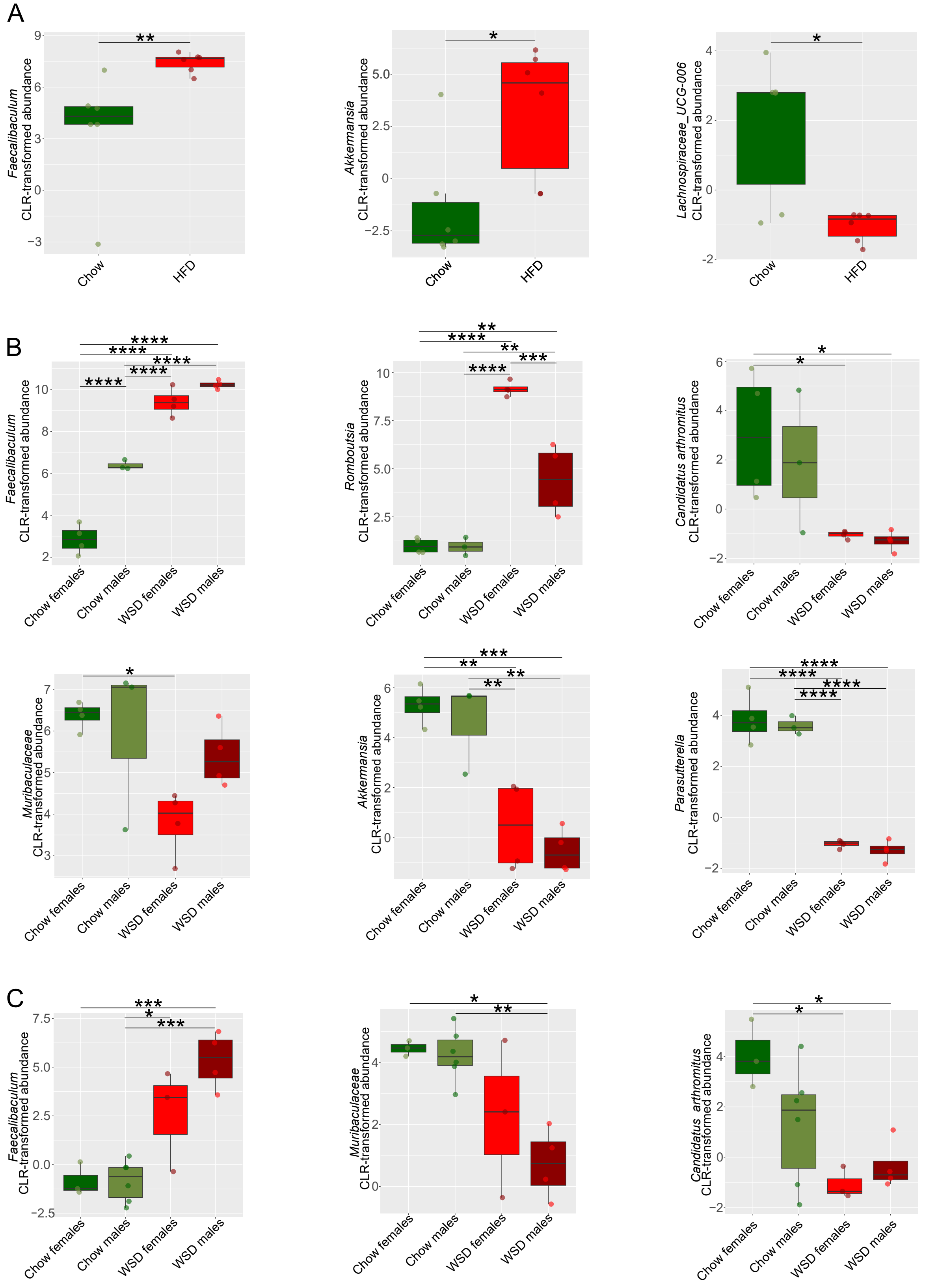

Supplementary Figure 4

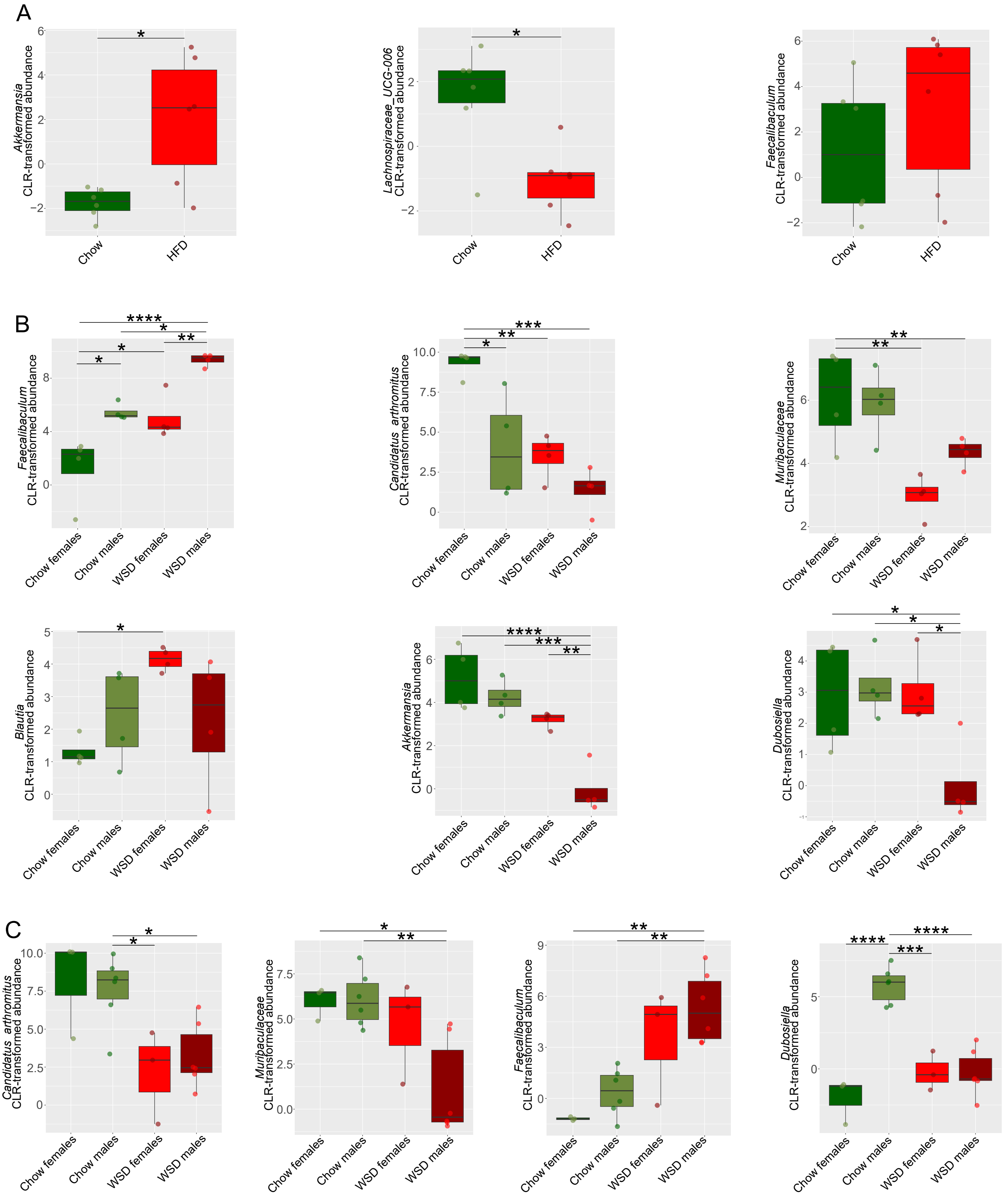

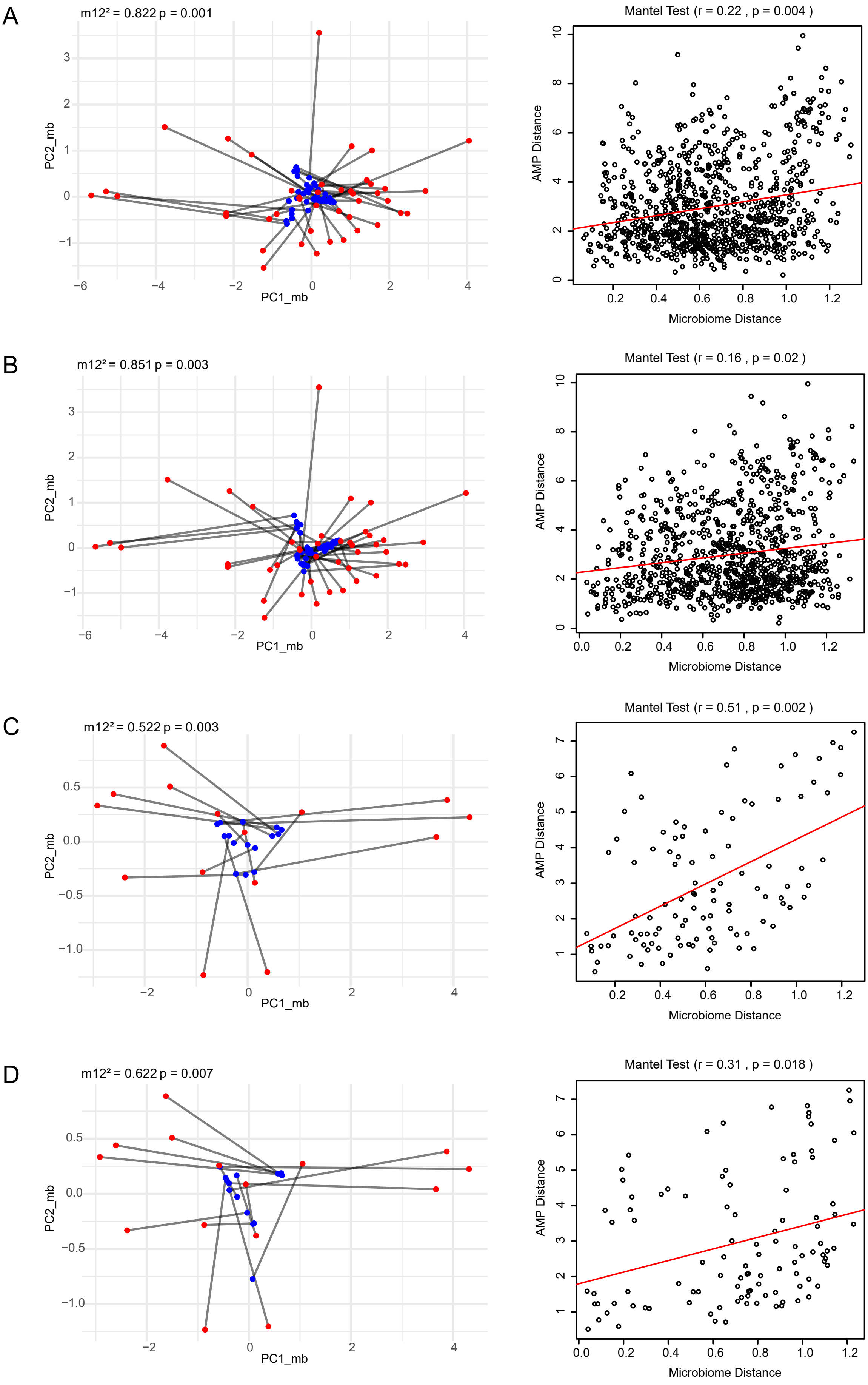
A
m12² = 0.822 p = 0.001
PC2_mb
PC1_mb
Mantel Test (r = 0.22 , p = 0.004 )
AMP Distance
Microbiome Distance
B
m12² = 0.851 p = 0.003
PC2_mb
PC1_mb
Mantel Test (r = 0.16 , p = 0.02 )
AMP Distance
Microbiome Distance
C
m12² = 0.522 p = 0.003
PC2_mb
PC1_mb
Mantel Test (r = 0.51 , p = 0.002 )
AMP Distance
Microbiome Distance
D
m12² = 0.622 p = 0.007
PC2_mb
PC1_mb
Mantel Test (r = 0.31 , p = 0.018 )
AMP Distance
Microbiome Distance
